# Supplementary material for: Continuous ammonia electrosynthesis using physically interlocked bipolar membrane at 1000 mA cm−2
Source: Nat Commun. 2023 Mar 23;14:1619. doi: 10.1038/s41467-023-37273-7 (PMC10036611; doi:10.1038/s41467-023-37273-7)
Supplement: Supplementary file 1 — Supplementary Information [file 41467_2023_37273_MOESM1_ESM.pdf]

Supplementary Information for

Continuous ammonia electrosynthesis using physically interlocked bipolar membrane at 1000 mA cm^{-2}

Ziang Xu[1], Lei Wan[1], Yiwen Liao[1], Maobin Pang[1], Qin Xu[1], Peican Wang[1] and Baoguo Wang[1]*

[1]Department of Chemical Engineering, Tsinghua University, Beijing, China

* Corresponding author: bgwang@tsinghua.edu.cn

Supplementary information contains

I. Supplementary Figures 2
II. Supplementary Tables 53
III. Supplementary Notes 57
IV. Supplementary References 76

I. Supplementary Figures

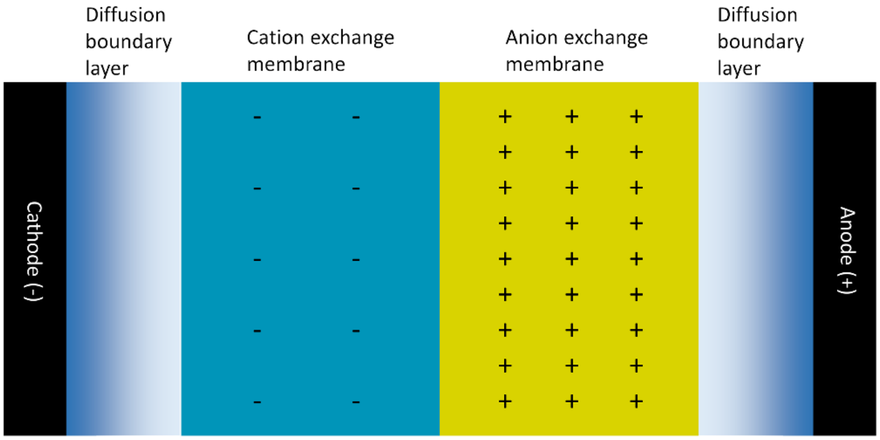

Supplementary Fig. 1 | Schematic illustration of one-dimensional bipolar membrane model for numerical simulation. Both the cation exchange layer and anion exchange layer are set to 50 μm with a 10 μm thickness of diffusion layer. The operating mode were set in reverse bias of bipolar membranes.

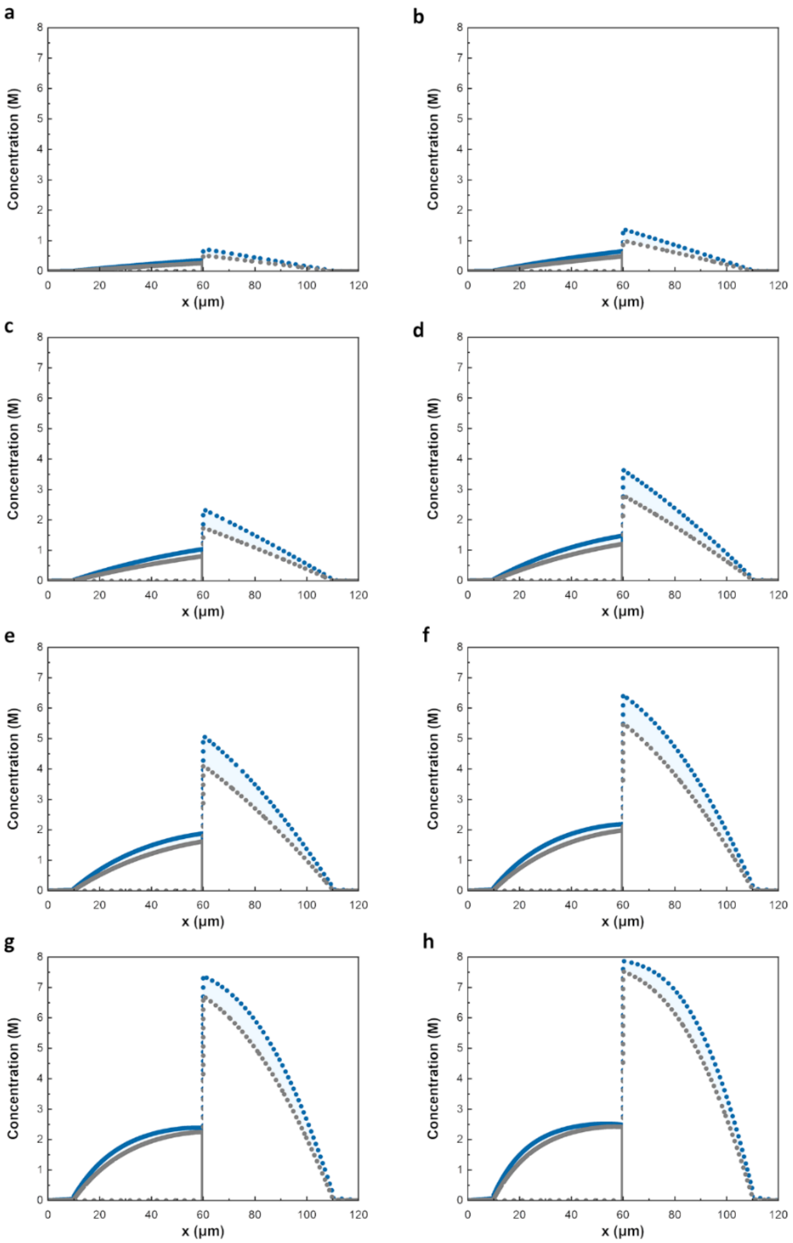

Supplementary Fig. 2 | All-range distribution of H^+ and OH^- concentration of normal bipolar membrane (gray line) and bipolar membrane with enhanced WD rate and ionic diffusion (mazarine) based on numerical simulation. The solid line and short dots represent for H^+ and OH^-, respectively. The applied voltage for bipolar membranes are **a,** 0.6 V; **b,** 0.7V; **c,** 0.8 V; **d,** 0.9 V; **e,** 1.0 V; **f,** 1.1 V; **g,** 1.2 V; **h,** 1.3 V.

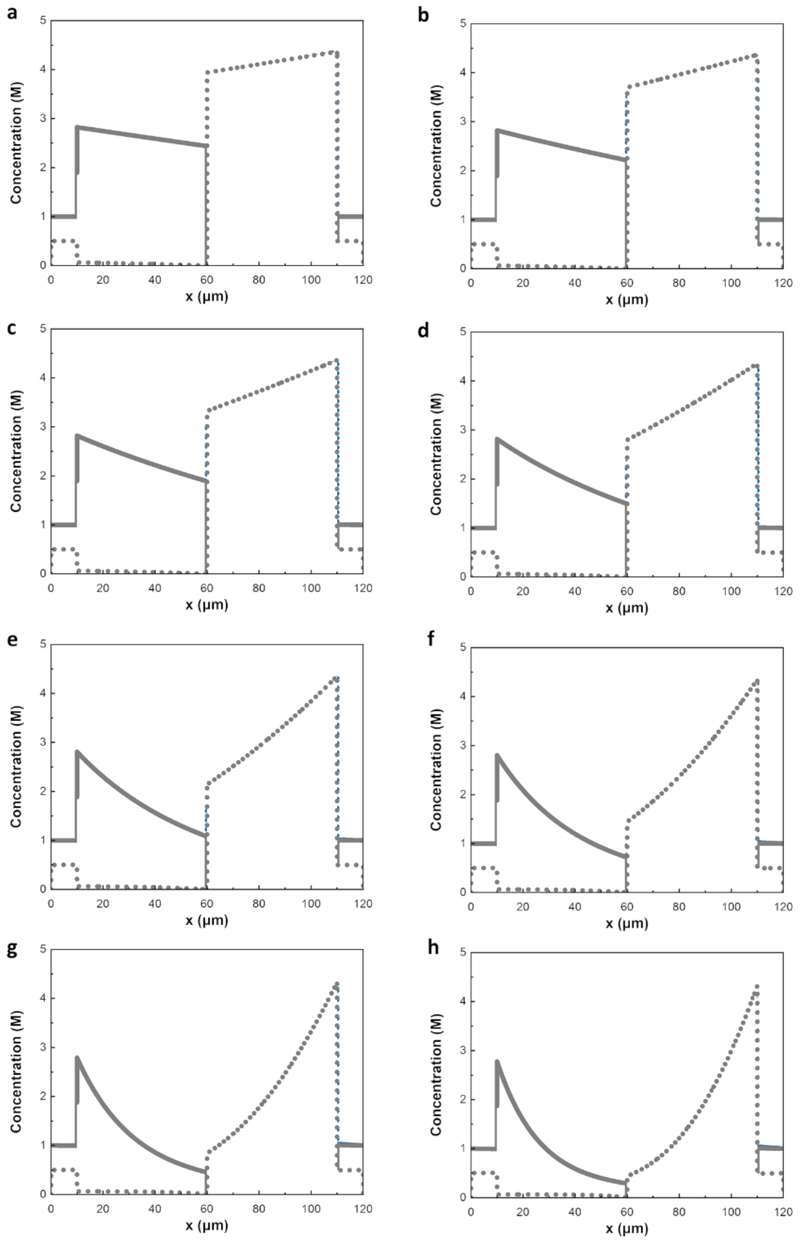

Supplementary Fig. 3 | Distribution of Na^{+} and SO_4^{-} concentration of normal bipolar membrane (gray line) and bipolar membrane with enhanced WD rate and ionic diffusion (mazarine) by numerical simulation. The solid line and short dots represent for Na^{+} and SO_4^{-}, respectively. The applied voltage for bipolar membranes are **a,** 0.6 V; **b,** 0.7V; **c,** 0.8 V; **d,** 0.9 V; **e,** 1.0 V; **f,** 1.1 V; **g,** 1.2 V; **h,** 1.3 V.

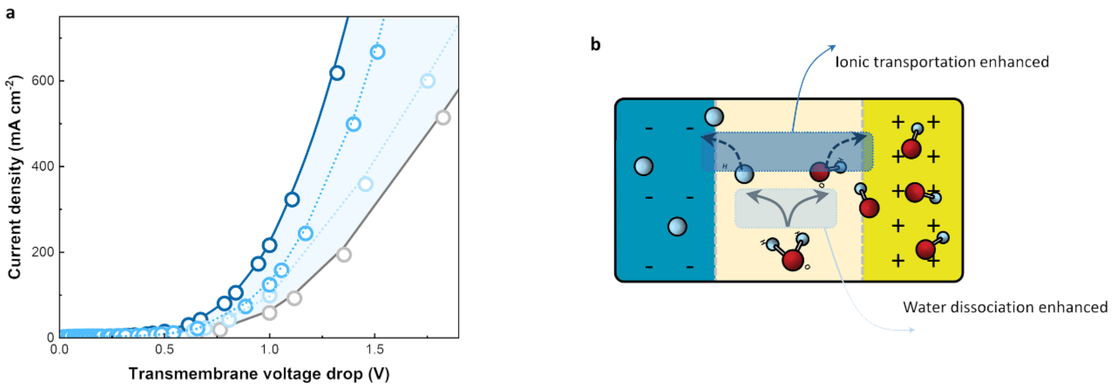

Supplementary Fig. 4. a, Numerical simulation results of current-voltage (I-V) curve for normal bipolar membrane (gray scatter-line), bipolar membrane with WD rate enhanced (wathet scatter-line), bipolar membrane with ionic diffusion enhanced (sky blue) and bipolar with both WD rate and ionic diffusion enhanced (mazarine). **b,** schematic illustration of total interfacial kinetic promotion leading by enhancing of WD rate and ionic transportation.

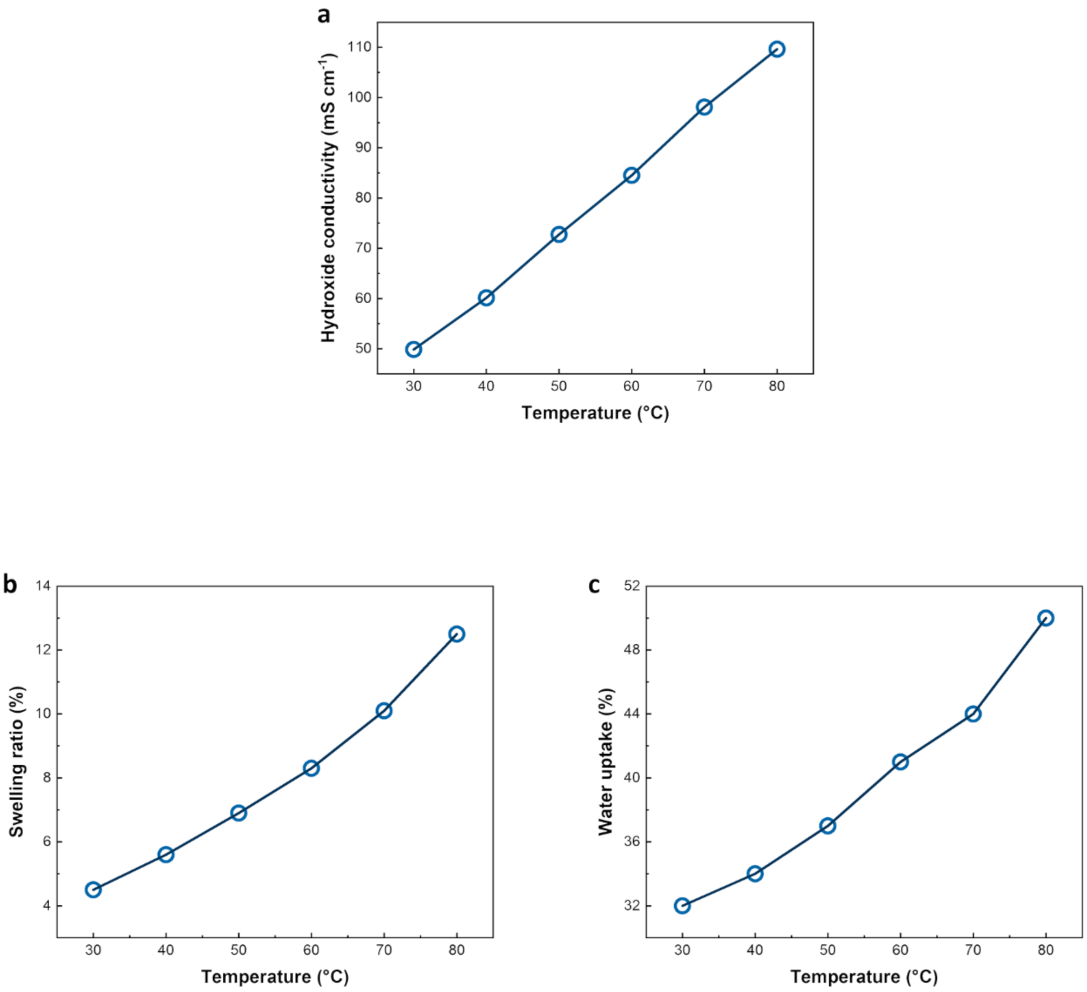

Supplementary Fig. 5 | Basic properties of QPPT as anion exchange layer vs. temperature. **a,** hydroxide conductivity; **b,** swelling ration (SR); **c,** water uptake (WU).

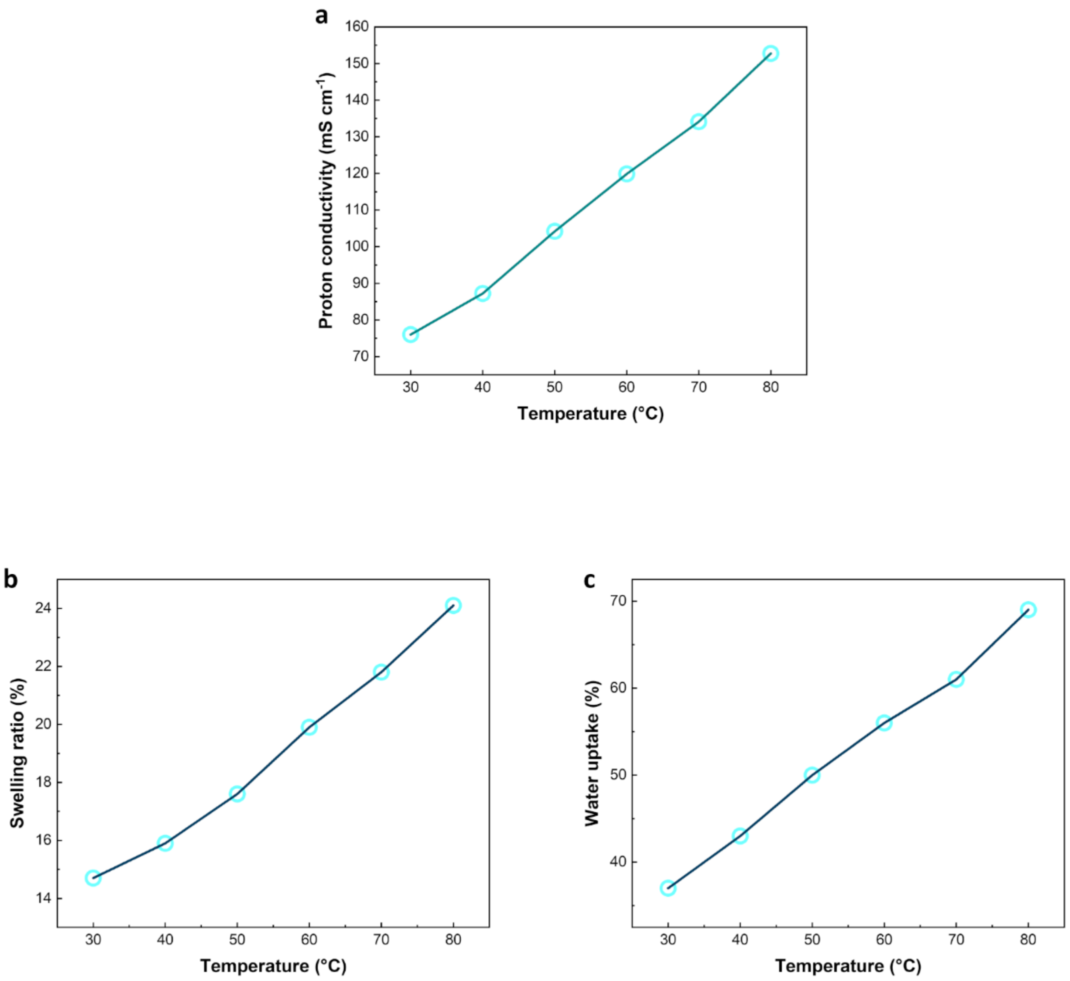

Supplementary Fig. 6 | Basic properties of PFSA as cation exchange layer vs. temperature. **a,** proton conductivity; **b,** swelling ration (SR); **c,** water uptake (WU).

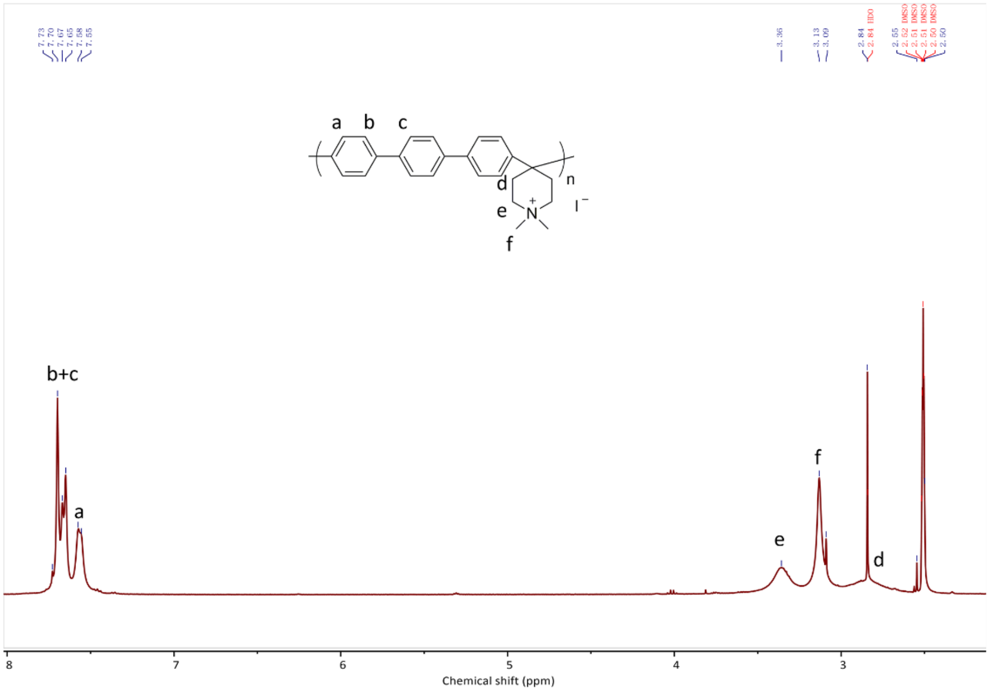

Supplementary Fig. 7 | ^{1}H NMR spectrum of Quaternary ammonia poly (N-methyl-piperidine-co-p-terphenyl) (QPPT) as anion exchange layer

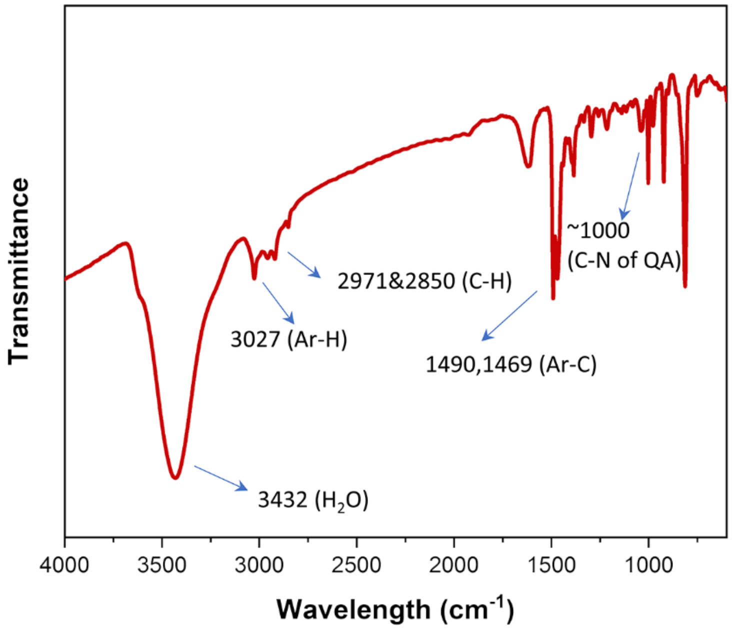

Supplementary Fig. 8 | FTIR spectrum of QPPT powder. Strong H_2O adsorption of the sample drying showed a hydrophilic nature of the polymer.

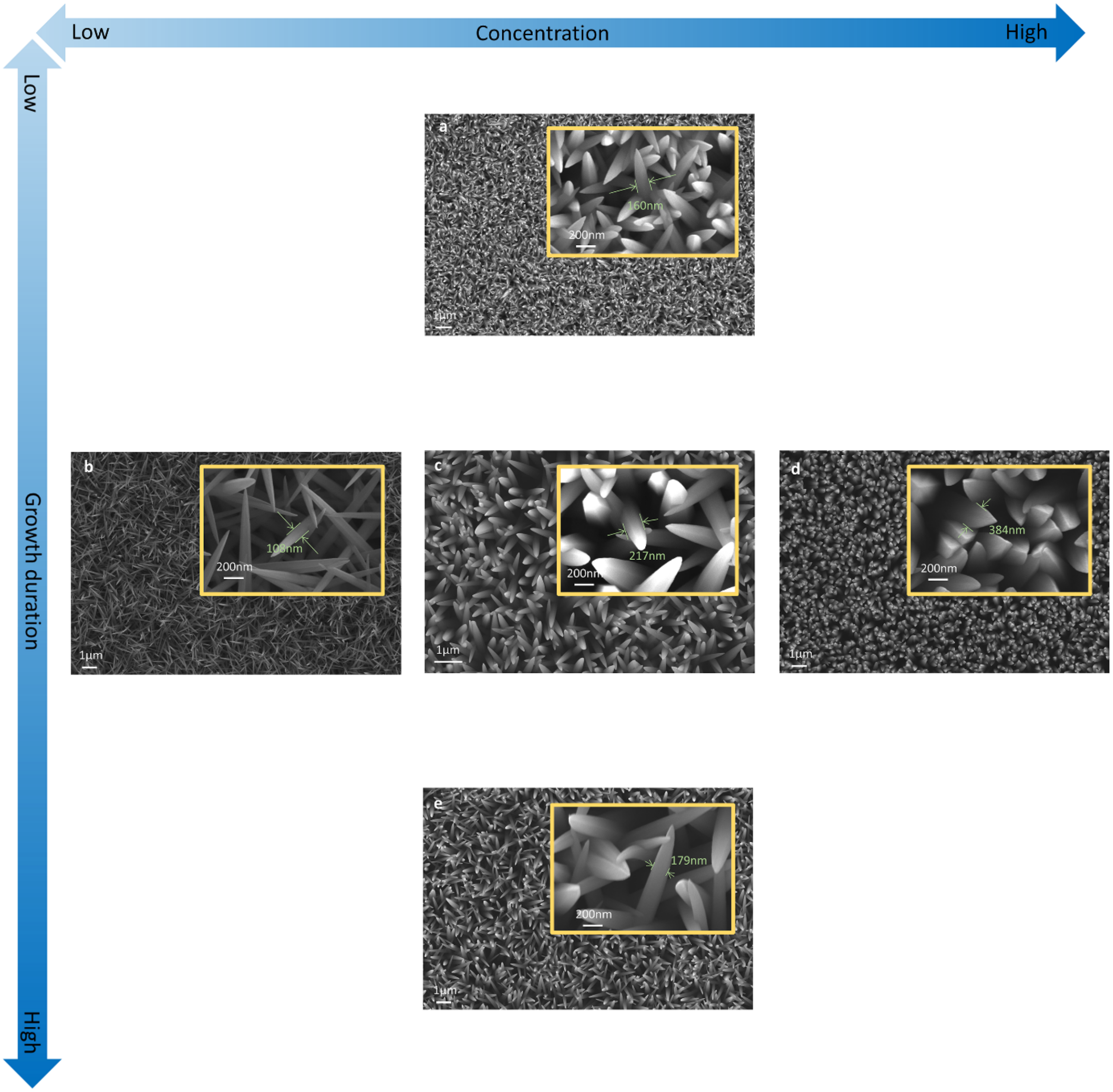

Supplementary Fig. 9 | Surface SEM images of CoNi templates with different synthesis conditions. **a,** precursor concentration: 3.2 wt% $CoCl_2·6H_2O$ & 1.6% $NiCl_2·6H_2O$; growth duration: 1.5 hours; **b,** precursor concentration: 0.64 wt% $CoCl_2·6H_2O$ & 0.32% $NiCl_2·6H_2O$; growth duration: 3 hours; **c,** precursor concentration: 3.2 wt% $CoCl_2·6H_2O$ & 1.6% $NiCl_2·6H_2O$; growth duration: 3 hours; **d,** precursor concentration: 6.4 wt% $CoCl_2·6H_2O$ & 3.2% $NiCl_2·6H_2O$; growth duration: 3 hours; **e,** precursor concentration: 3.2 wt% $CoCl_2·6H_2O$ & 1.6% $NiCl_2·6H_2O$; growth duration: 4.5 hours. The insets of each figures show a magnified image.

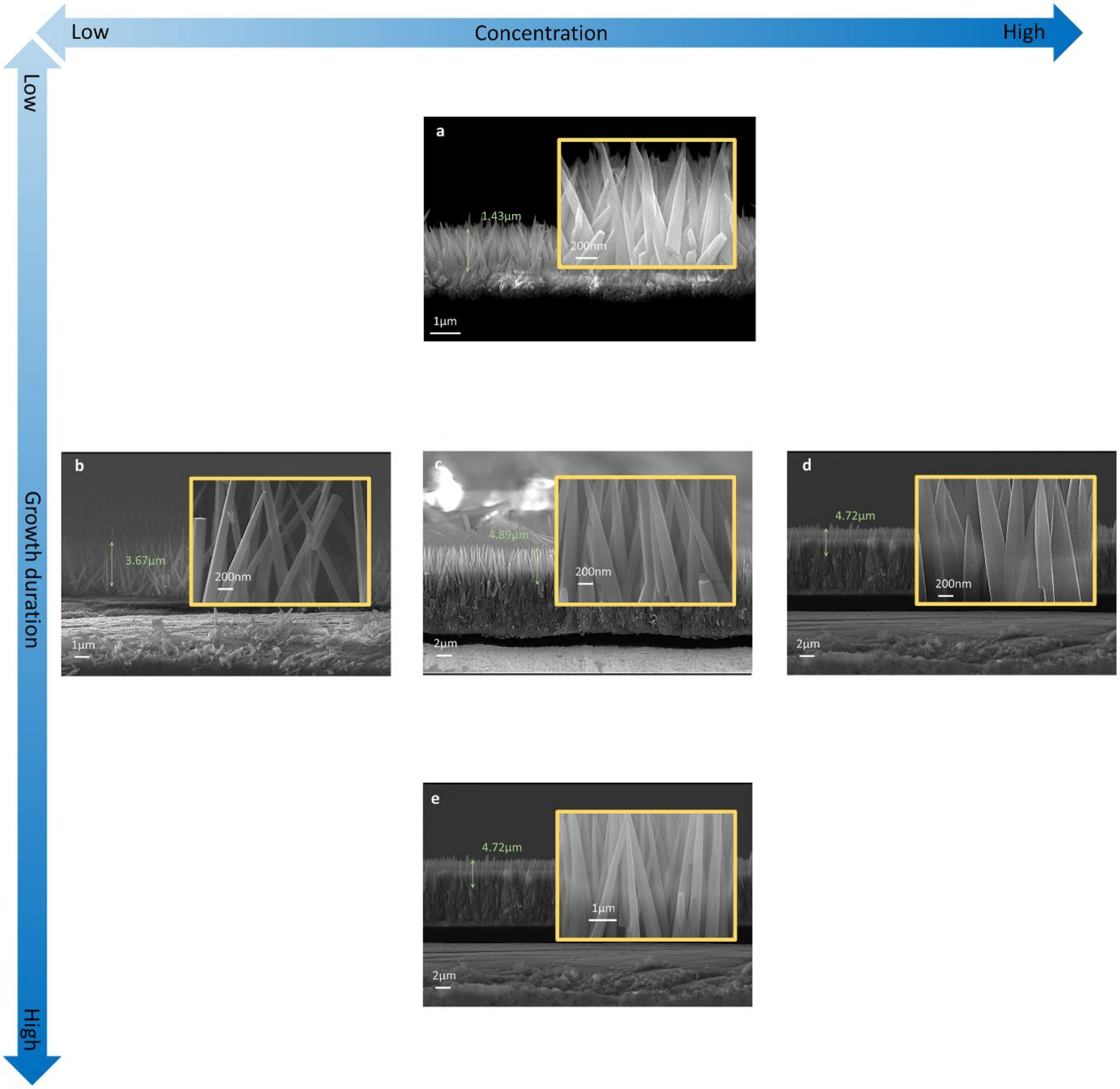

Supplementary Fig. 10 | Cross-section SEM images of CoNi templates with different synthesis conditions. **a,** precursor concentration: 3.2 wt% $CoCl_2{\cdot}6H_2O$ & 1.6% $NiCl_2{\cdot}6H_2O$; growth duration: 1.5 hours; **b,** precursor concentration: 0.64 wt% $CoCl_2{\cdot}6H_2O$ & 0.32% $NiCl_2{\cdot}6H_2O$; growth duration: 3 hours; **c,** precursor concentration: 3.2 wt% $CoCl_2{\cdot}6H_2O$ & 1.6% $NiCl_2{\cdot}6H_2O$; growth duration: 3 hours; **d,** precursor concentration: 6.4 wt% $CoCl_2{\cdot}6H_2O$ & 3.2% $NiCl_2{\cdot}6H_2O$; growth duration: 3 hours; **e,** precursor concentration: 3.2 wt% $CoCl_2{\cdot}6H_2O$ & 1.6% $NiCl_2{\cdot}6H_2O$; growth duration: 4.5 hours. The insets of each figures show a magnified image.

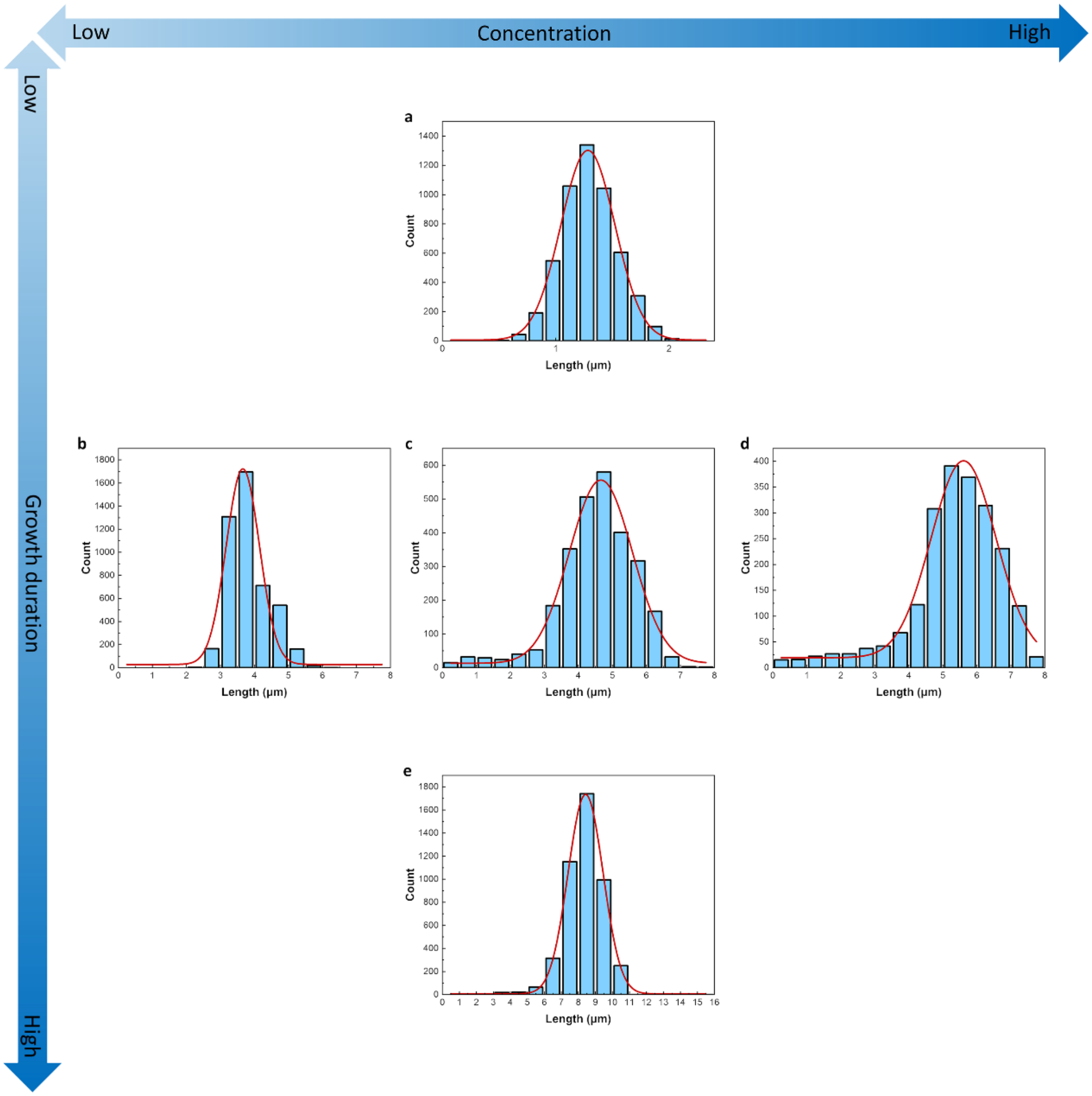

Supplementary Fig. 11 | Height frequency of CoNi nano array grow on Ni substrate based on Gaussian distribution. **a,** precursor concentration: 3.2 wt% $CoCl_2·6H_2O$ & 1.6% $NiCl_2·6H_2O$; growth duration: 1.5 hours; **b,** precursor concentration: 0.64 wt% $CoCl_2·6H_2O$ & 0.32% $NiCl_2·6H_2O$; growth duration: 3 hours; **c,** precursor concentration: 3.2 wt% $CoCl_2·6H_2O$ & 1.6% $NiCl_2·6H_2O$; growth duration: 3 hours; **d,** precursor concentration: 6.4 wt% $CoCl_2·6H_2O$ & 3.2% $NiCl_2·6H_2O$; growth duration: 3 hours; **e,** precursor concentration: 3.2 wt% $CoCl_2·6H_2O$ & 1.6% $NiCl_2·6H_2O$; growth duration: 4.5 hours.

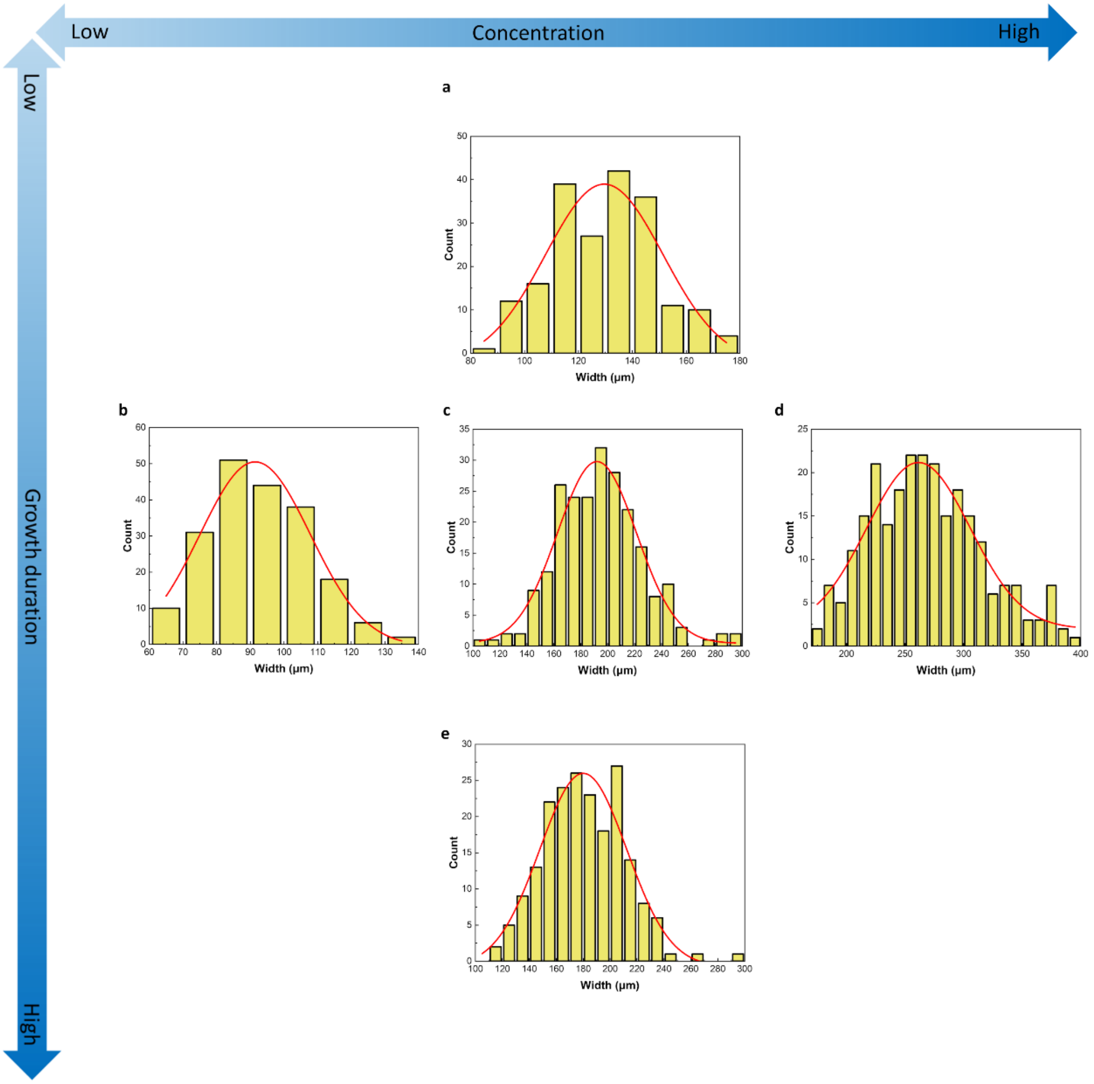

Supplementary Fig. 12 | Width frequency of CoNi nano array grow on Ni substrate based on Gaussian distribution. **a,** precursor concentration: 3.2 wt% $CoCl_2 \cdot 6H_2O$ & 1.6% $NiCl_2 \cdot 6H_2O$; growth duration: 1.5 hours; **b,** precursor concentration: 0.64 wt% $CoCl_2 \cdot 6H_2O$ & 0.32% $NiCl_2 \cdot 6H_2O$; growth duration: 3 hours; **c,** precursor concentration: 3.2 wt% $CoCl_2 \cdot 6H_2O$ & 1.6% $NiCl_2 \cdot 6H_2O$; growth duration: 3 hours; **d,** precursor concentration: 6.4 wt% $CoCl_2 \cdot 6H_2O$ & 3.2% $NiCl_2 \cdot 6H_2O$; growth duration: 3 hours; **e,** precursor concentration: 3.2 wt% $CoCl_2 \cdot 6H_2O$ & 1.6% $NiCl_2 \cdot 6H_2O$; growth duration: 4.5 hours.

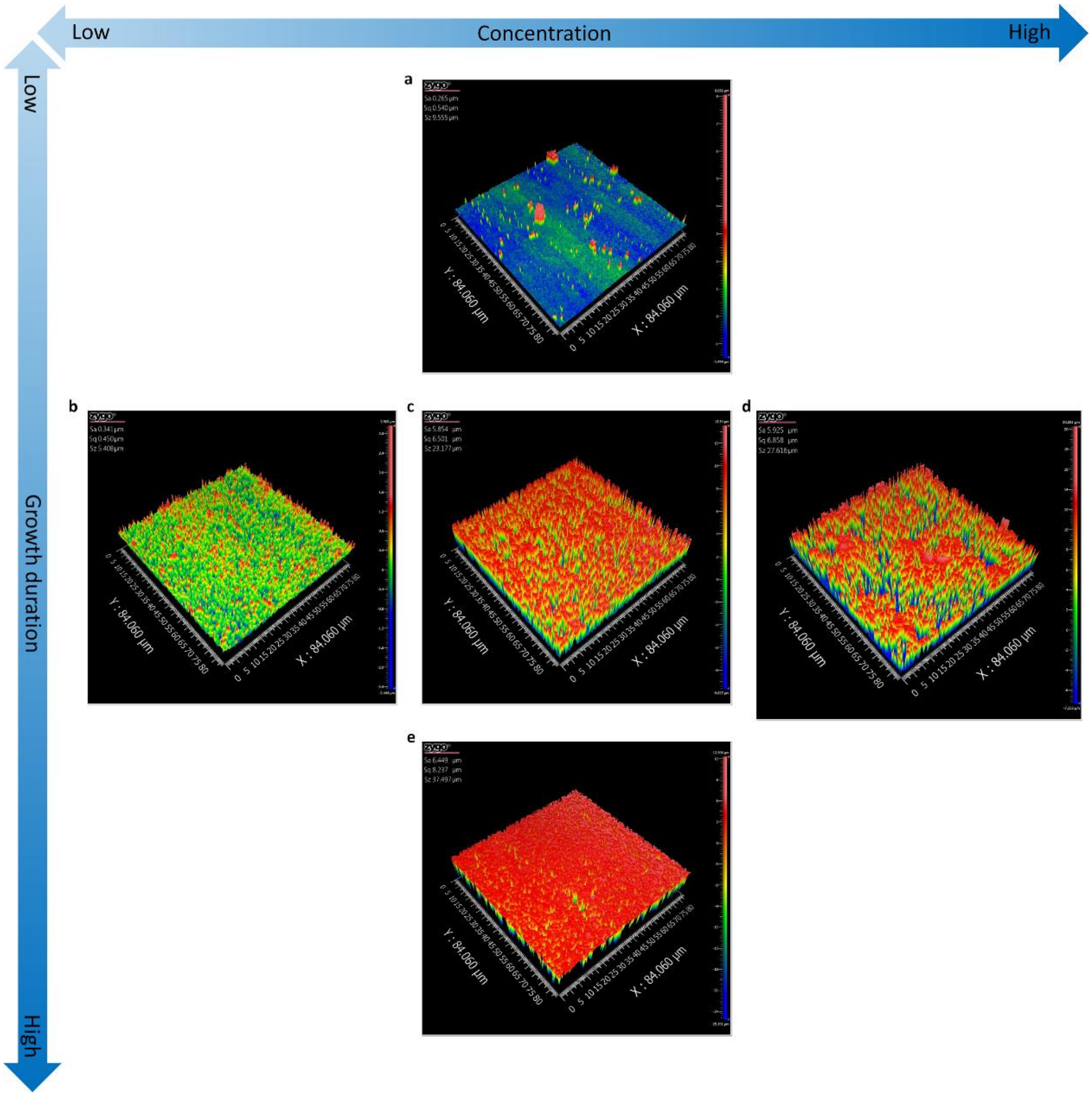

Supplementary Fig. 13 | Surface topography of CoNi nano array grow on Ni substrate based on three-dimensional white light interference method **a,** precursor concentration: 3.2 wt% $CoCl_2·6H_2O$ & 1.6% $NiCl_2·6H_2O$; growth duration: 1.5 hours; **b,** precursor concentration: 0.64 wt% $CoCl_2·6H_2O$ & 0.32% $NiCl_2·6H_2O$; growth duration: 3 hours; **c,** precursor concentration: 3.2 wt% $CoCl_2·6H_2O$ & 1.6% $NiCl_2·6H_2O$; growth duration: 3 hours; **d,** precursor concentration: 6.4 wt% $CoCl_2·6H_2O$ & 3.2% $NiCl_2·6H_2O$; growth duration: 3 hours; **e,** precursor concentration: 3.2 wt% $CoCl_2·6H_2O$ & 1.6% $NiCl_2·6H_2O$; growth duration: 4.5 hours.

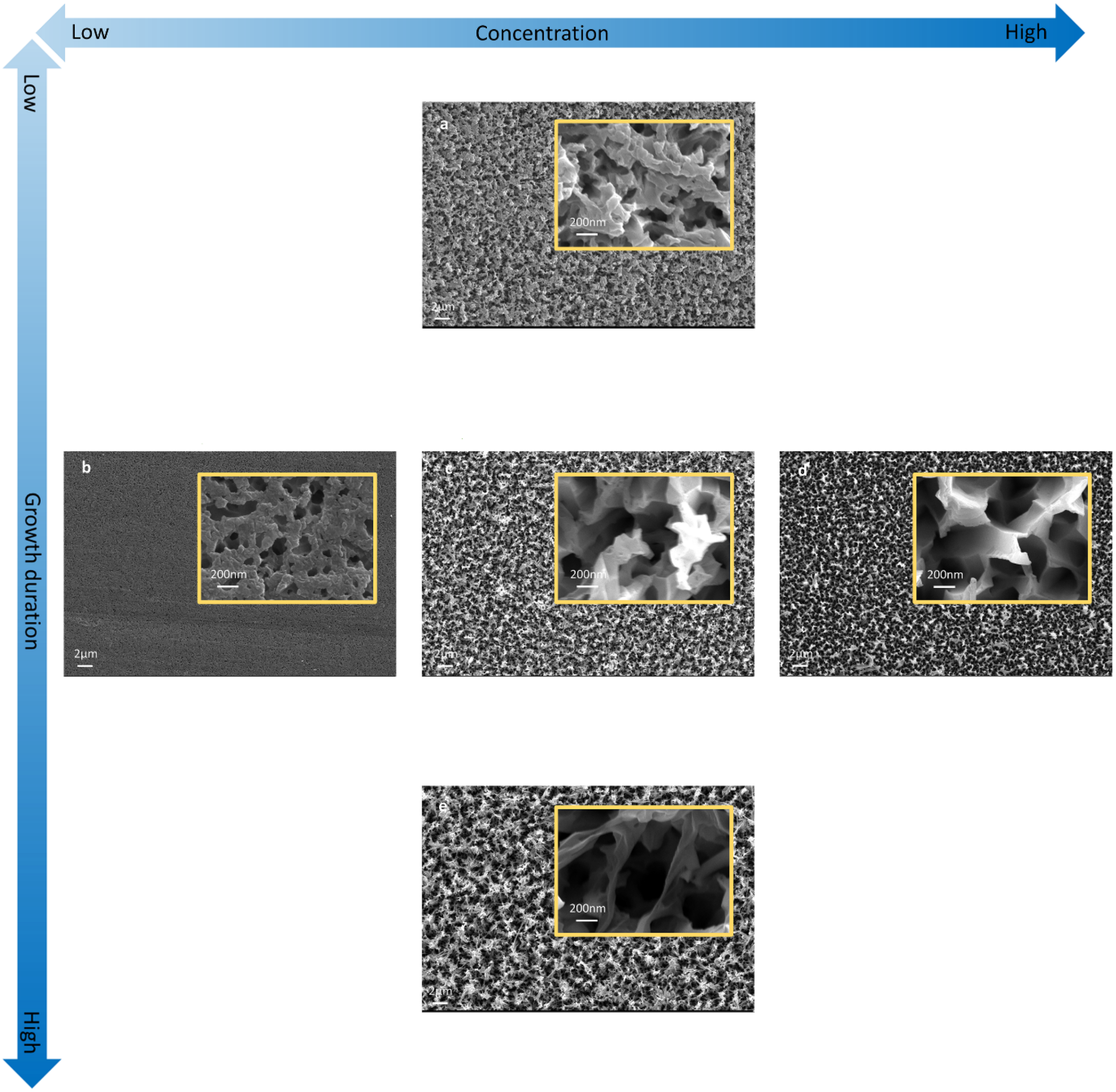

Supplementary Fig. 14 | Surface SEM images of AEM transferred with different templates. **a,** precursor concentration: 3.2 wt% $CoCl_2{\cdot}6H_2O$ & 1.6% $NiCl_2{\cdot}6H_2O$; growth duration: 1.5 hours; **b,** precursor concentration: 0.64 wt% $CoCl_2{\cdot}6H_2O$ & 0.32% $NiCl_2{\cdot}6H_2O$; growth duration: 3 hours; **c,** precursor concentration: 3.2 wt% $CoCl_2{\cdot}6H_2O$ & 1.6% $NiCl_2{\cdot}6H_2O$; growth duration: 3 hours; **d,** precursor concentration: 6.4 wt% $CoCl_2{\cdot}6H_2O$ & 3.2% $NiCl_2{\cdot}6H_2O$; growth duration: 3 hours; **e,** precursor concentration: 3.2 wt% $CoCl_2{\cdot}6H_2O$ & 1.6% $NiCl_2{\cdot}6H_2O$; growth duration: 4.5 hours. The insets of each figures show a magnified image.

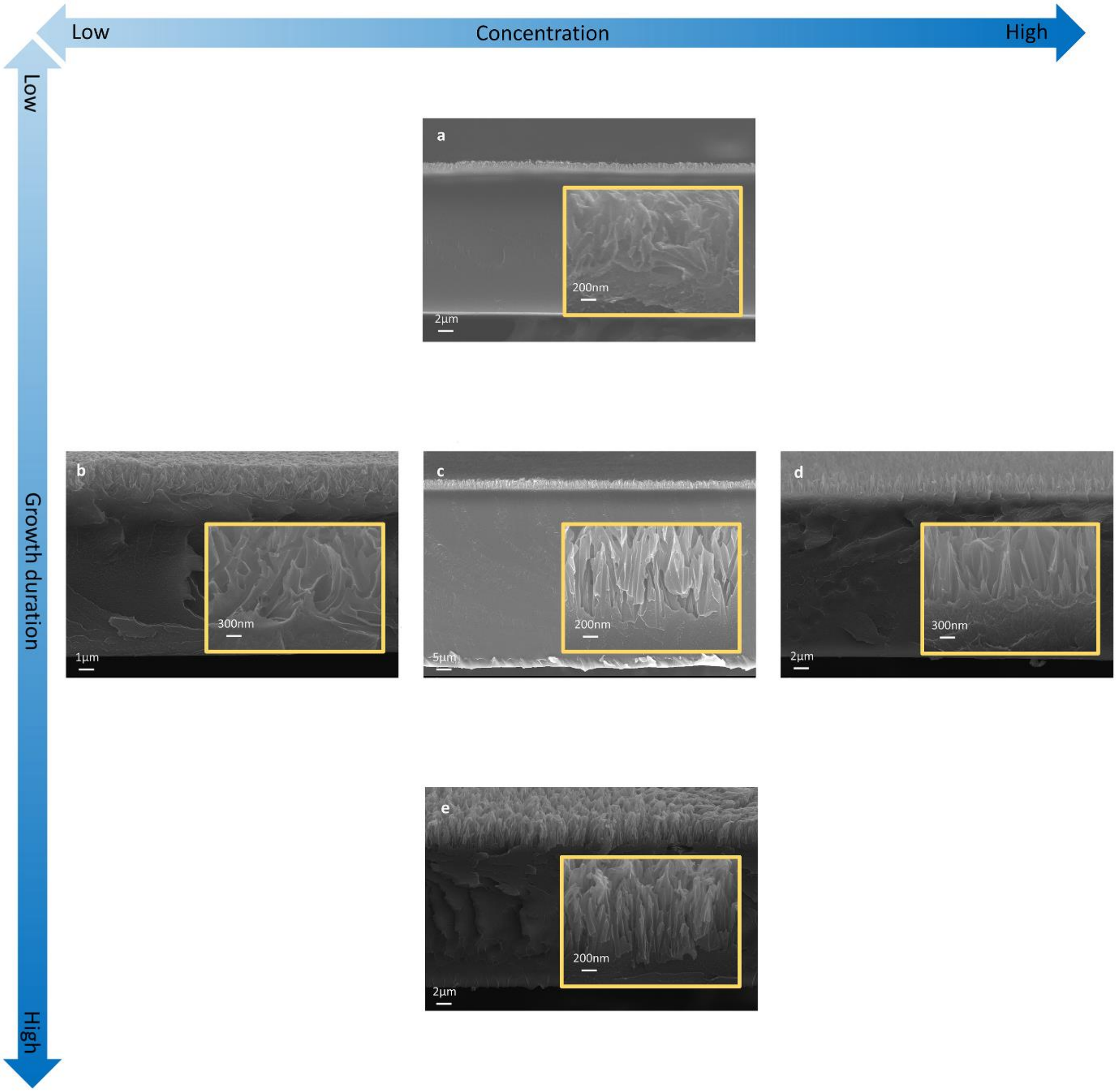

Supplementary Fig. 15 | Cross-section SEM images of AEM transferred with different templates. **a,** precursor concentration: 3.2 wt% $CoCl_2·6H_2O$ & 1.6% $NiCl_2·6H_2O$; growth duration: 1.5 hours; **b,** precursor concentration: 0.64 wt% $CoCl_2·6H_2O$ & 0.32% $NiCl_2·6H_2O$; growth duration: 3 hours; **c,** precursor concentration: 3.2 wt% $CoCl_2·6H_2O$ & 1.6% $NiCl_2·6H_2O$; growth duration: 3 hours; **d,** precursor concentration: 6.4 wt% $CoCl_2·6H_2O$ & 3.2% $NiCl_2·6H_2O$; growth duration: 3 hours; **e,** precursor concentration: 3.2 wt% $CoCl_2·6H_2O$ & 1.6% $NiCl_2·6H_2O$; growth duration: 4.5 hours. The insets of each figures show a magnified image.

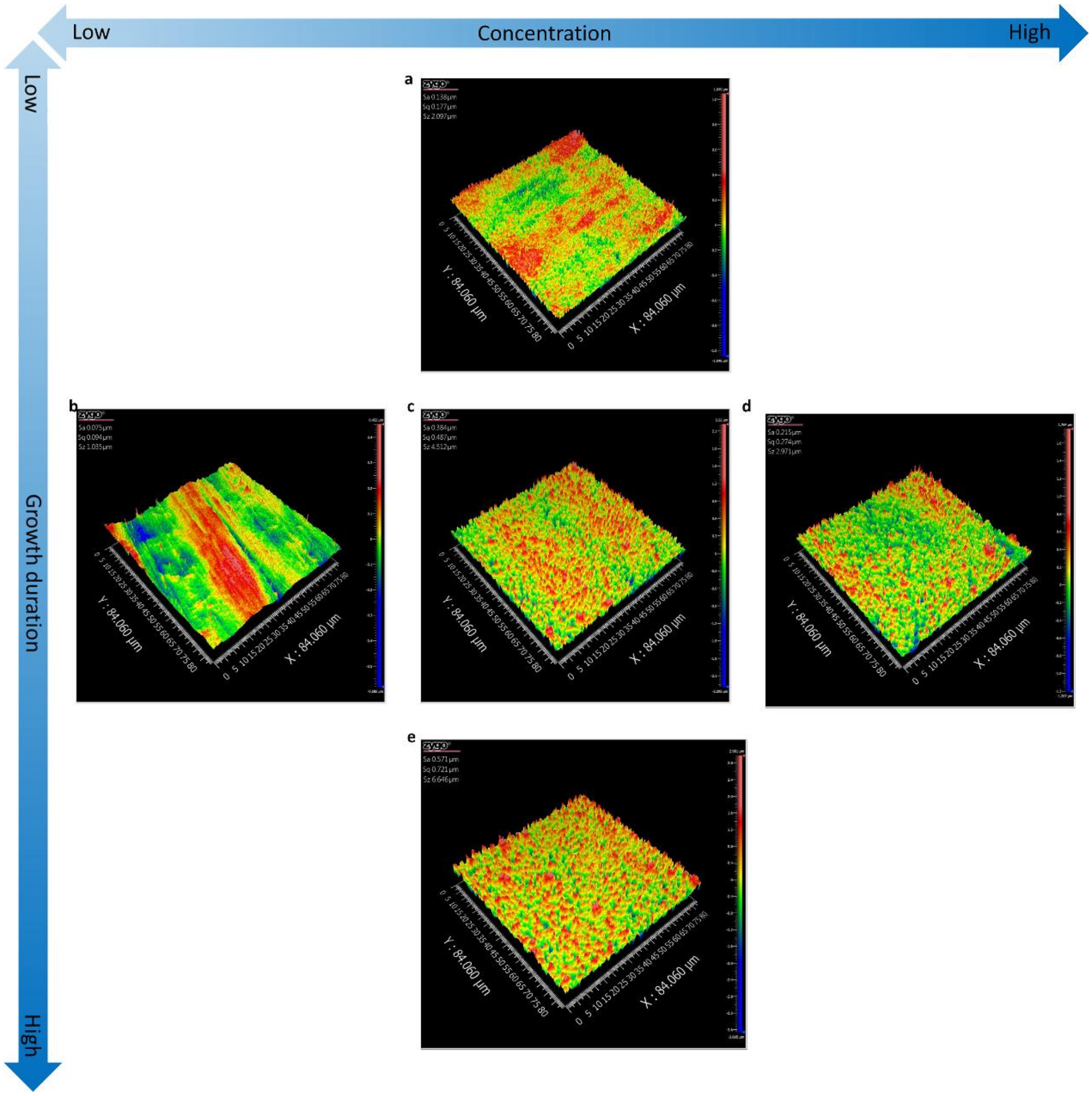

Supplementary Fig. 16 | Surface topography of anion exchange layer by template-transferred based on three-dimensional white light interference method **a,** precursor concentration: 3.2 wt% $CoCl_2{\cdot}6H_2O$ & 1.6% $NiCl_2{\cdot}6H_2O$; growth duration: 1.5 hours; **b,** precursor concentration: 0.64 wt% $CoCl_2{\cdot}6H_2O$ & 0.32% $NiCl_2{\cdot}6H_2O$; growth duration: 3 hours; **c,** precursor concentration: 3.2 wt% $CoCl_2{\cdot}6H_2O$ & 1.6% $NiCl_2{\cdot}6H_2O$; growth duration: 3 hours; **d,** precursor concentration: 6.4 wt% $CoCl_2{\cdot}6H_2O$ & 3.2% $NiCl_2{\cdot}6H_2O$; growth duration: 3 hours; **e,** precursor concentration: 3.2 wt% $CoCl_2{\cdot}6H_2O$ & 1.6% $NiCl_2{\cdot}6H_2O$; growth duration: 4.5 hours.

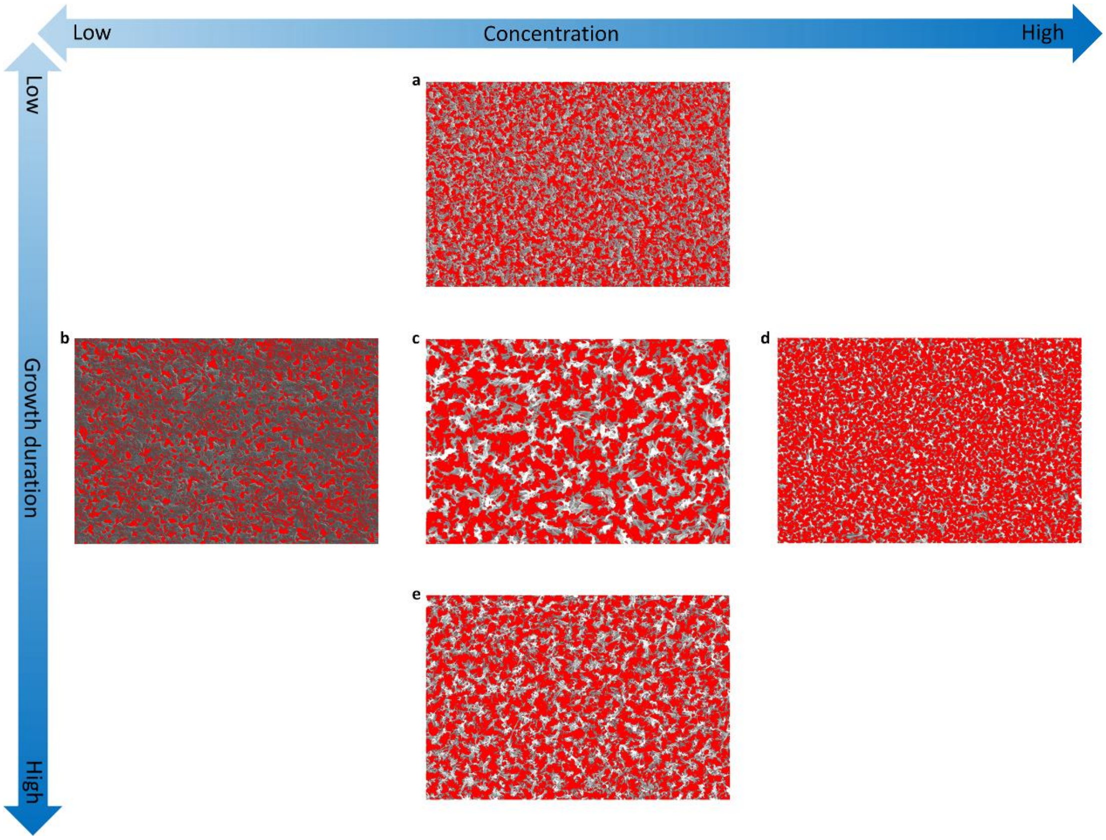

Supplementary Fig. 17 | Surface areal porosity determination of anion exchange layer by template-transferred by ImageJ software (gray scale identification) **a,** precursor concentration: 3.2 wt% $CoCl_2 \cdot 6H_2O$ & 1.6% $NiCl_2 \cdot 6H_2O$; growth duration: 1.5 hours; **b,** precursor concentration: 0.64 wt% $CoCl_2 \cdot 6H_2O$ & 0.32% $NiCl_2 \cdot 6H_2O$; growth duration: 3 hours; **c,** precursor concentration: 3.2 wt% $CoCl_2 \cdot 6H_2O$ & 1.6% $NiCl_2 \cdot 6H_2O$; growth duration: 3 hours; **d,** precursor concentration: 6.4 wt% $CoCl_2 \cdot 6H_2O$ & 3.2% $NiCl_2 \cdot 6H_2O$; growth duration: 3 hours; **e,** precursor concentration: 3.2 wt% $CoCl_2 \cdot 6H_2O$ & 1.6% $NiCl_2 \cdot 6H_2O$; growth duration: 4.5 hours.

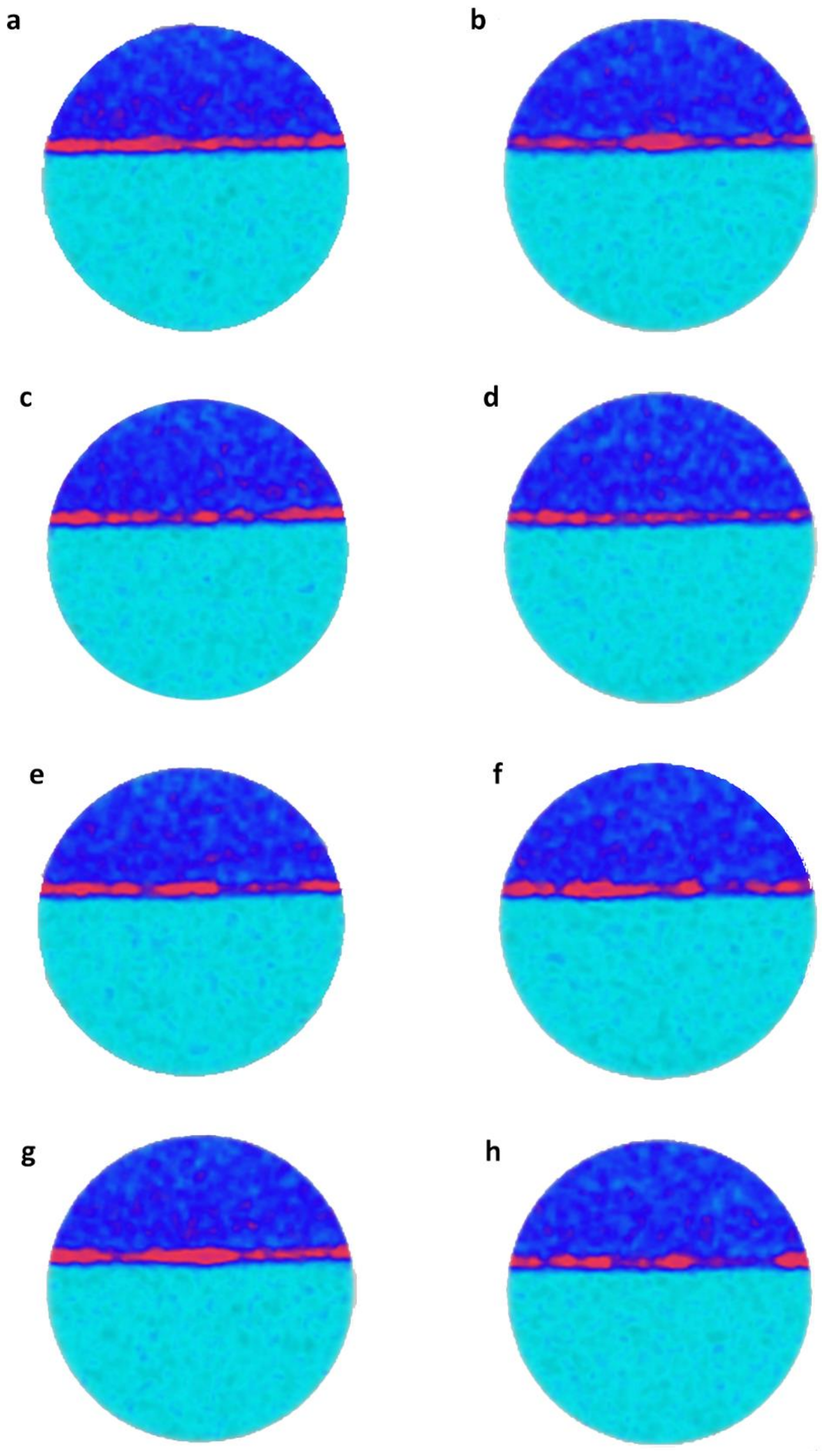

Supplementary Fig. 18 | Snapshots of 3D reconstruction projection of MBM at various depth. **a,** 0 μm; **b,** 2 μm; **c,** 4 μm; **d,** 6 μm; **e,** 8 μm; **f,** 10 μm; **g,** 12 μm; **h,** 14 μm.

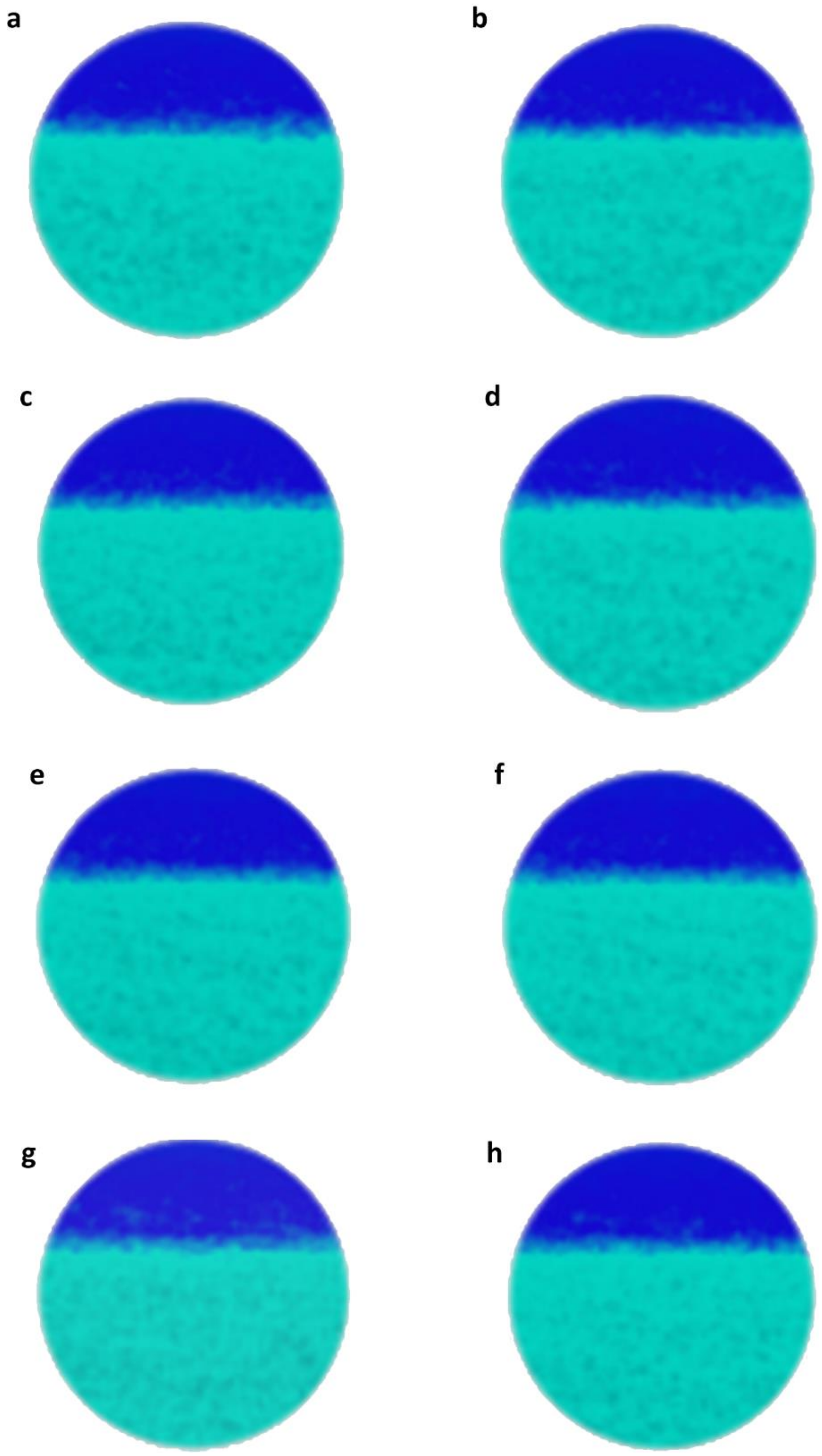

Supplementary Fig. 19 | Snapshots of 3D reconstruction projection of FBM at various depth. **a,** 0 μm; **b,** 2 μm; **c,** 4 μm; **d,** 6 μm; **e,** 8 μm; **f,** 10 μm; **g,** 12 μm; **h,** 14 μm.

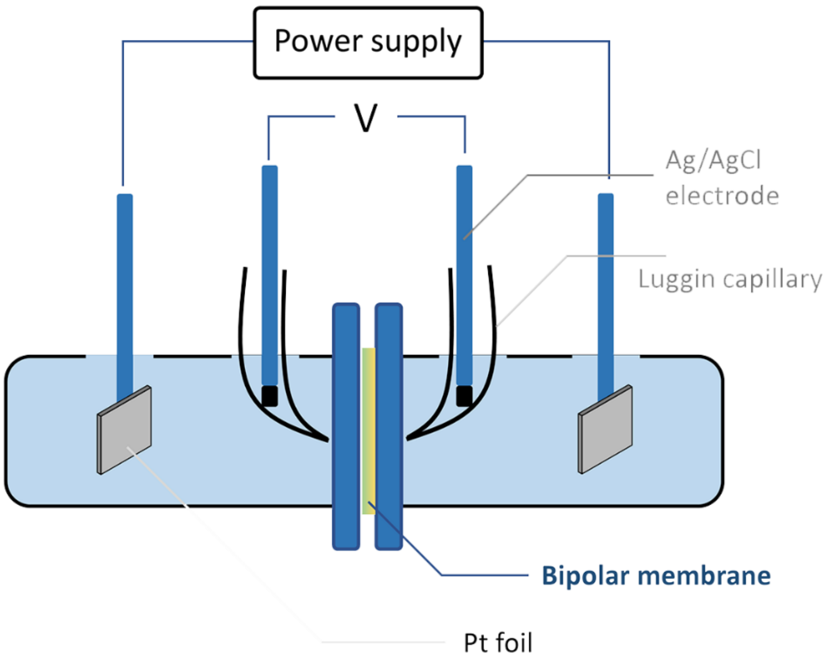

Supplementary Fig. 20 | Schematic illustration of four-electrode measurements setup for I-V relationship, long-term WD stability and EIS measurements

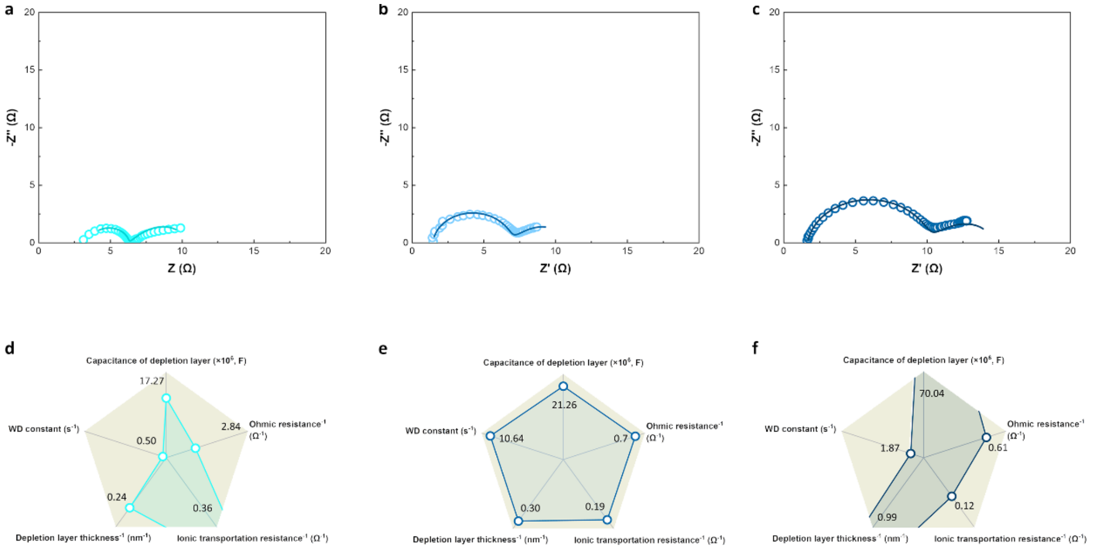

Supplementary Fig. 21 | EIS measurements (**a-c**) and parameter fitting results (**d-f**) of MBM(s) at 5 mA cm^{-2}. **a, d,** precursor concentration: 3.2 wt% $CoCl_2 \cdot 6H_2O$ & 1.6% $NiCl_2 \cdot 6H_2O$; growth duration: 1.5 hours; **b, e,** precursor concentration: 3.2 wt% $CoCl_2 \cdot 6H_2O$ & 1.6% $NiCl_2 \cdot 6H_2O$; growth duration: 3 hours; **c, f,** precursor concentration: 3.2 wt% $CoCl_2 \cdot 6H_2O$ & 1.6% $NiCl_2 \cdot 6H_2O$; growth duration: 4.5 hours.

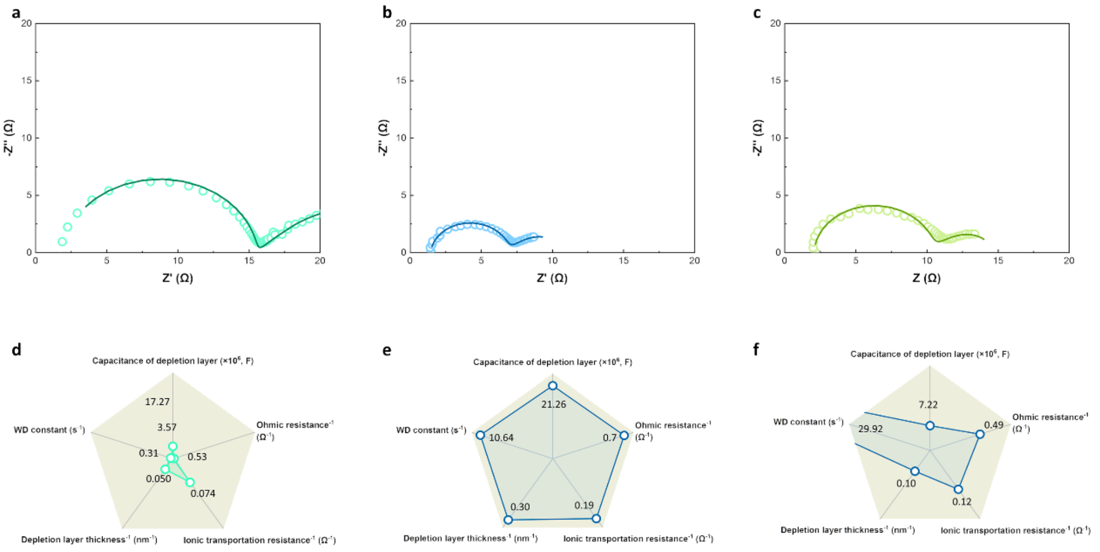

Supplementary Fig. 22 | EIS measurements (**a-c**) and parameter fitting results (**d-f**) of MBM(s) at 5 mA cm^{-2}. **a, d,** precursor concentration: 0.64 wt% $CoCl_2{\cdot}6H_2O$ & 0.32% $NiCl_2{\cdot}6H_2O$; growth duration: 3 hours; **b, e,** precursor concentration: 3.2 wt% $CoCl_2{\cdot}6H_2O$ & 1.6% $NiCl_2{\cdot}6H_2O$; growth duration: 3 hours; **c, f,** precursor concentration: 6.4 wt% $CoCl_2{\cdot}6H_2O$ & 3.2% $NiCl_2{\cdot}6H_2O$; growth duration: 3 hours.

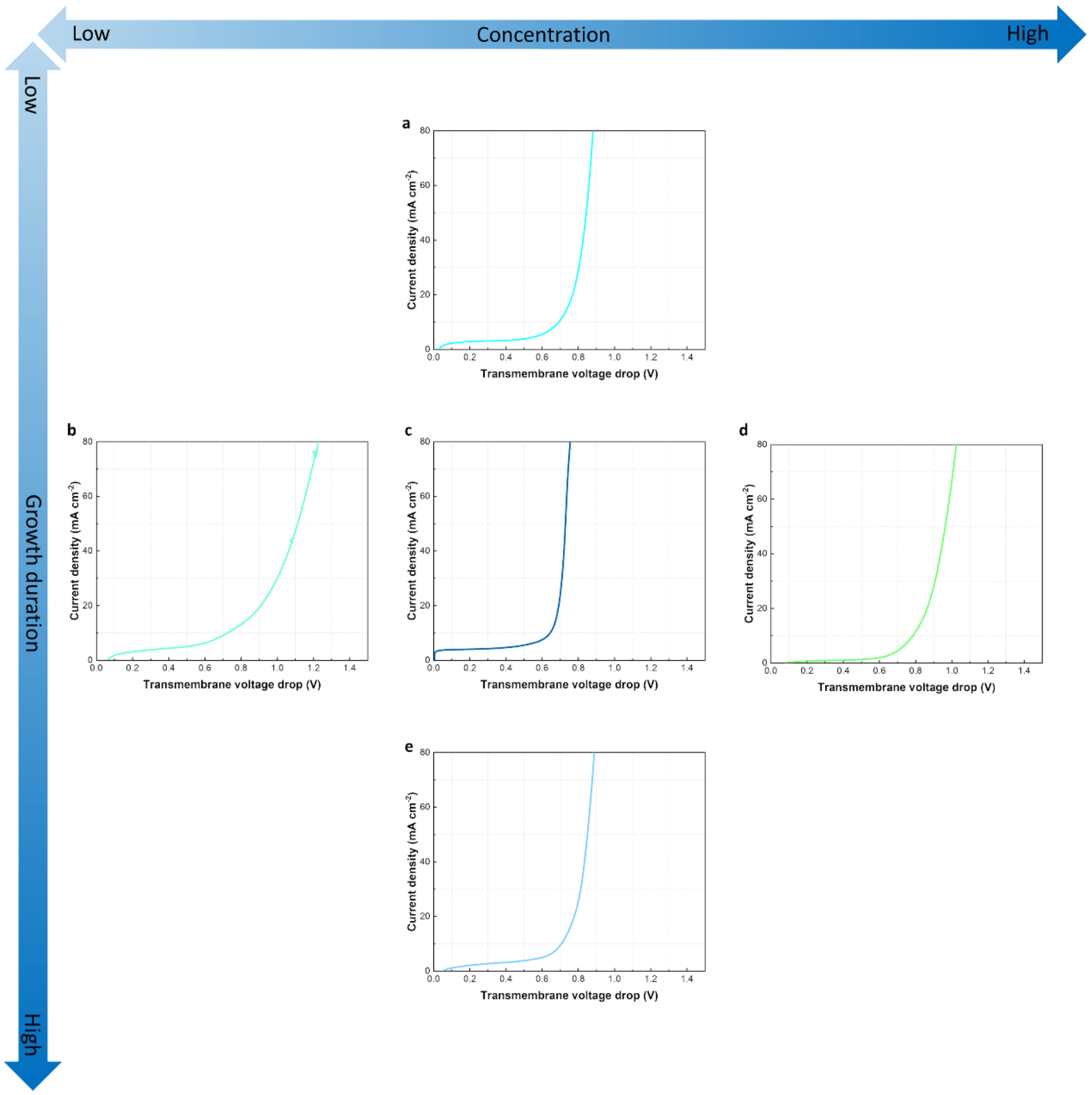

Supplementary Fig. 23 | I-V relationships of MBM(s) at current density range from 0 to 80 mA cm^{-2} constructed with AEL structure transferred with templates: **a,** precursor concentration: 3.2 wt% $CoCl_2·6H_2O$ & 1.6% $NiCl_2·6H_2O$; growth duration: 1.5 hours; **b,** precursor concentration: 0.64 wt% $CoCl_2·6H_2O$ & 0.32% $NiCl_2·6H_2O$; growth duration: 3 hours; **c,** precursor concentration: 3.2 wt% $CoCl_2·6H_2O$ & 1.6% $NiCl_2·6H_2O$; growth duration: 3 hours; **d,** precursor concentration: 6.4 wt% $CoCl_2·6H_2O$ & 3.2% $NiCl_2·6H_2O$; growth duration: 3 hours; **e,** precursor concentration: 3.2 wt% $CoCl_2·6H_2O$ & 1.6% $NiCl_2·6H_2O$; growth duration: 4.5 hours.

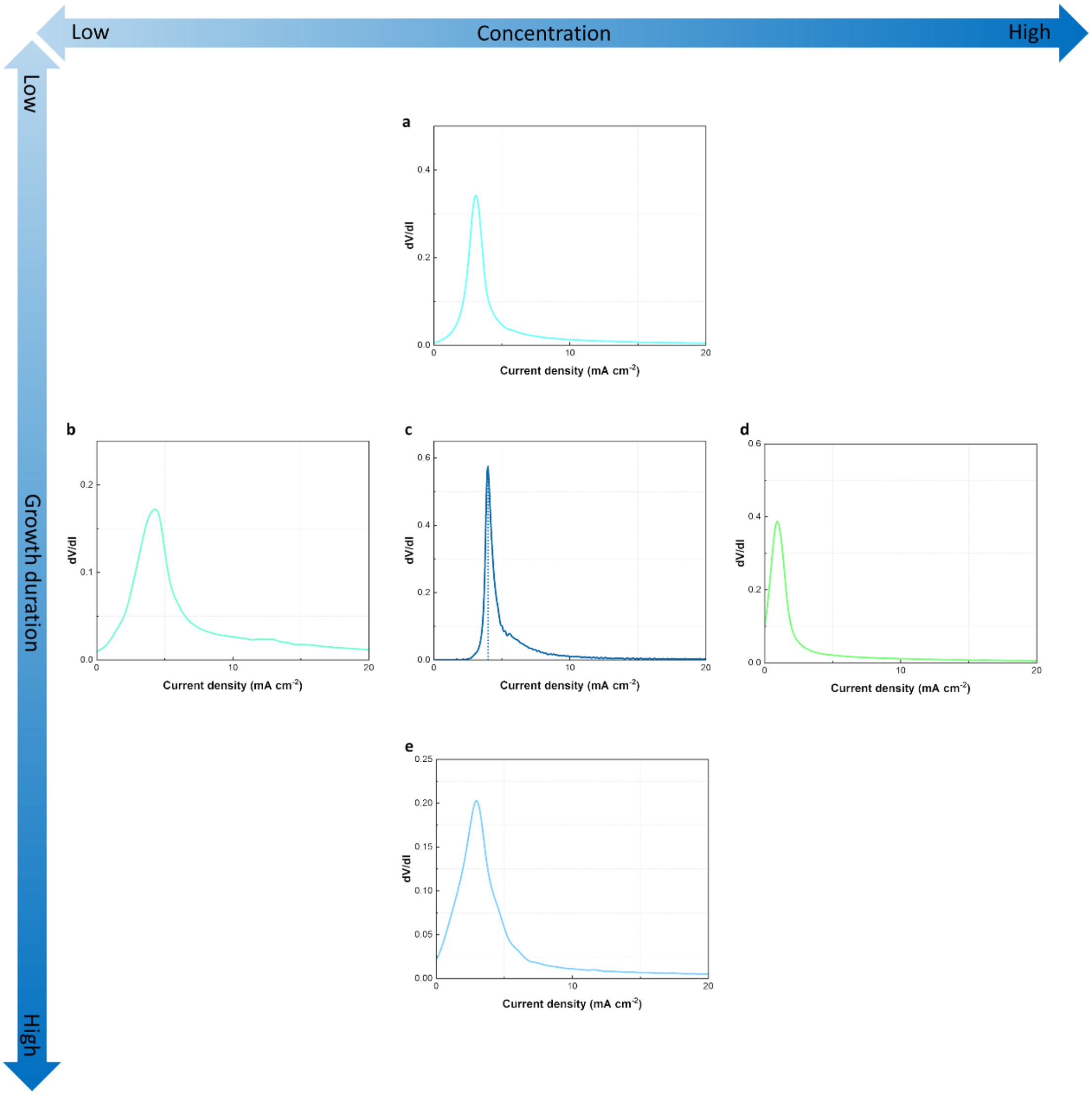

Supplementary Fig. 24 | 1st limiting current density (derived from dV/dI vs. current density) of MBM(s) constructed with AEL structure transferred with templates: **a,** precursor concentration: 3.2 wt% $CoCl_2 \cdot 6H_2O$ & 1.6% $NiCl_2 \cdot 6H_2O$; growth duration: 1.5 hours; **b,** precursor concentration: 0.64 wt% $CoCl_2 \cdot 6H_2O$ & 0.32% $NiCl_2 \cdot 6H_2O$; growth duration: 3 hours; **c,** precursor concentration: 3.2 wt% $CoCl_2 \cdot 6H_2O$ & 1.6% $NiCl_2 \cdot 6H_2O$; growth duration: 3 hours; **d,** precursor concentration: 6.4 wt% $CoCl_2 \cdot 6H_2O$ & 3.2% $NiCl_2 \cdot 6H_2O$; growth duration: 3 hours; **e,** precursor concentration: 3.2 wt% $CoCl_2 \cdot 6H_2O$ & 1.6% $NiCl_2 \cdot 6H_2O$; growth duration: 4.5 hours.

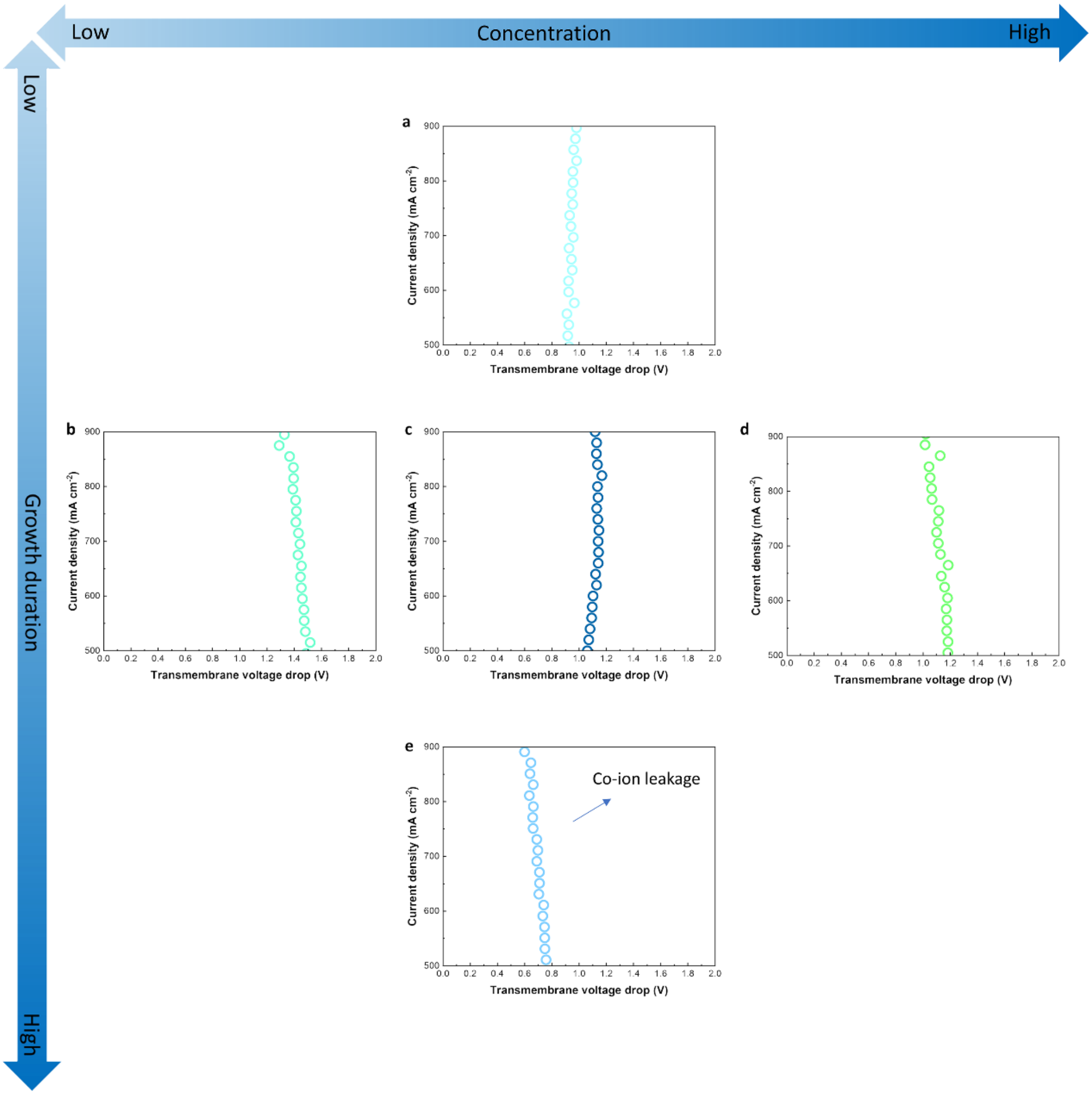

Supplementary Fig. 25 | I-V relationships of MBM(s) at current density range from 500 mA cm^{-2} to 900 mA cm^{-2} constructed with AEL structure transferred with templates: **a,** precursor concentration: 3.2 wt% $CoCl_2 \cdot 6H_2O$ & 1.6% $NiCl_2 \cdot 6H_2O$; growth duration: 1.5 hours; **b,** precursor concentration: 0.64 wt% $CoCl_2 \cdot 6H_2O$ & 0.32% $NiCl_2 \cdot 6H_2O$; growth duration: 3 hours; **c,** precursor concentration: 3.2 wt% $CoCl_2 \cdot 6H_2O$ & 1.6% $NiCl_2 \cdot 6H_2O$; growth duration: 3 hours; **d,** precursor concentration: 6.4 wt% $CoCl_2 \cdot 6H_2O$ & 3.2% $NiCl_2 \cdot 6H_2O$; growth duration: 3 hours; **e,** precursor concentration: 3.2 wt% $CoCl_2 \cdot 6H_2O$ & 1.6% $NiCl_2 \cdot 6H_2O$; growth duration: 4.5 hours.

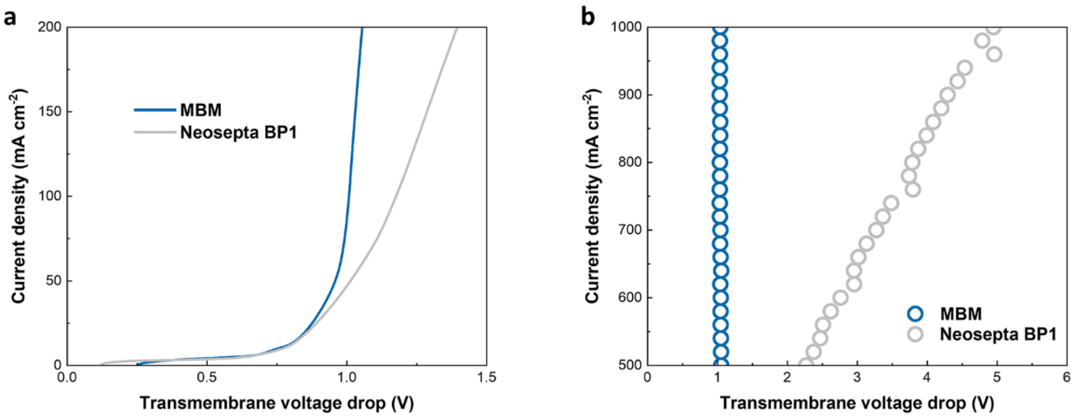

Supplementary Fig. 26 | Water dissociation I-V curves of MBM and commercial Neosepta BP1 in alkaline medium (1 M KOH). **a,** 0-200 mA cm^{-2}; **b,** 500-1000 mA cm^{-2}.

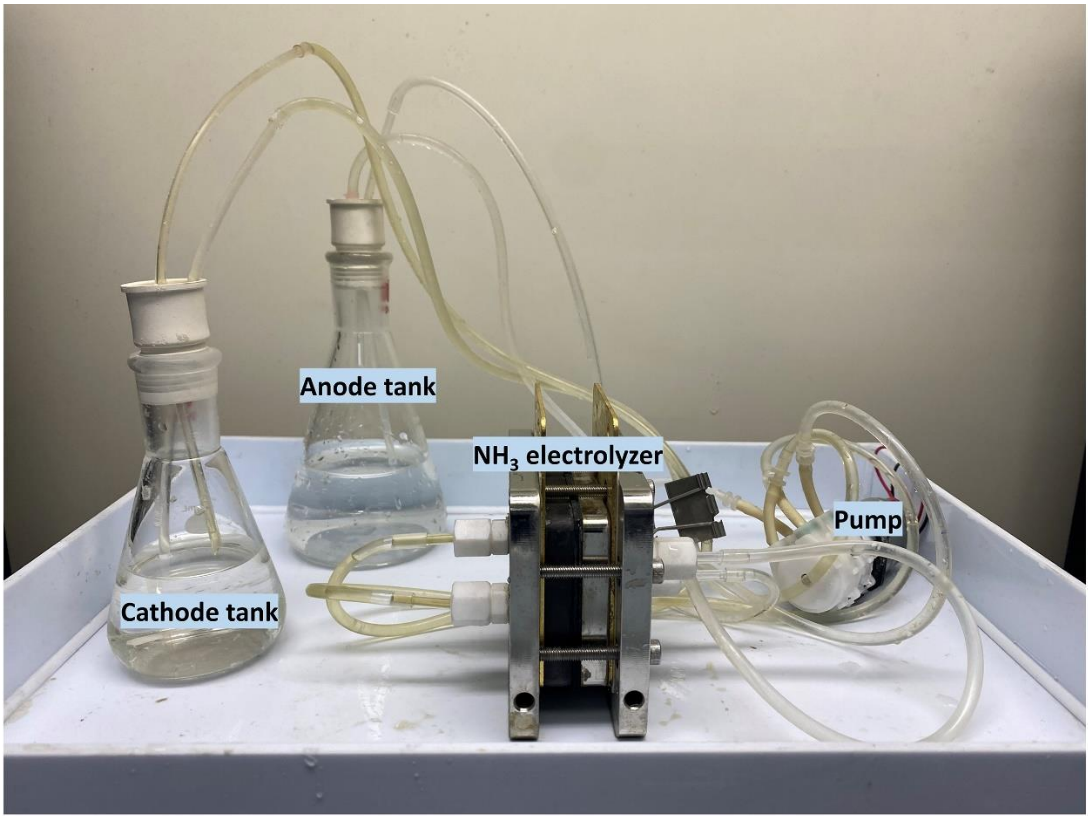

Supplementary Fig. 27 | Photography description of continuous NH_3 electrosynthesis flow cell system. The electrolytes were 1 M KOH/2000 ppm KNO_3 in cathode tank and is 1 M KOH in anode tank before startup, respectively.

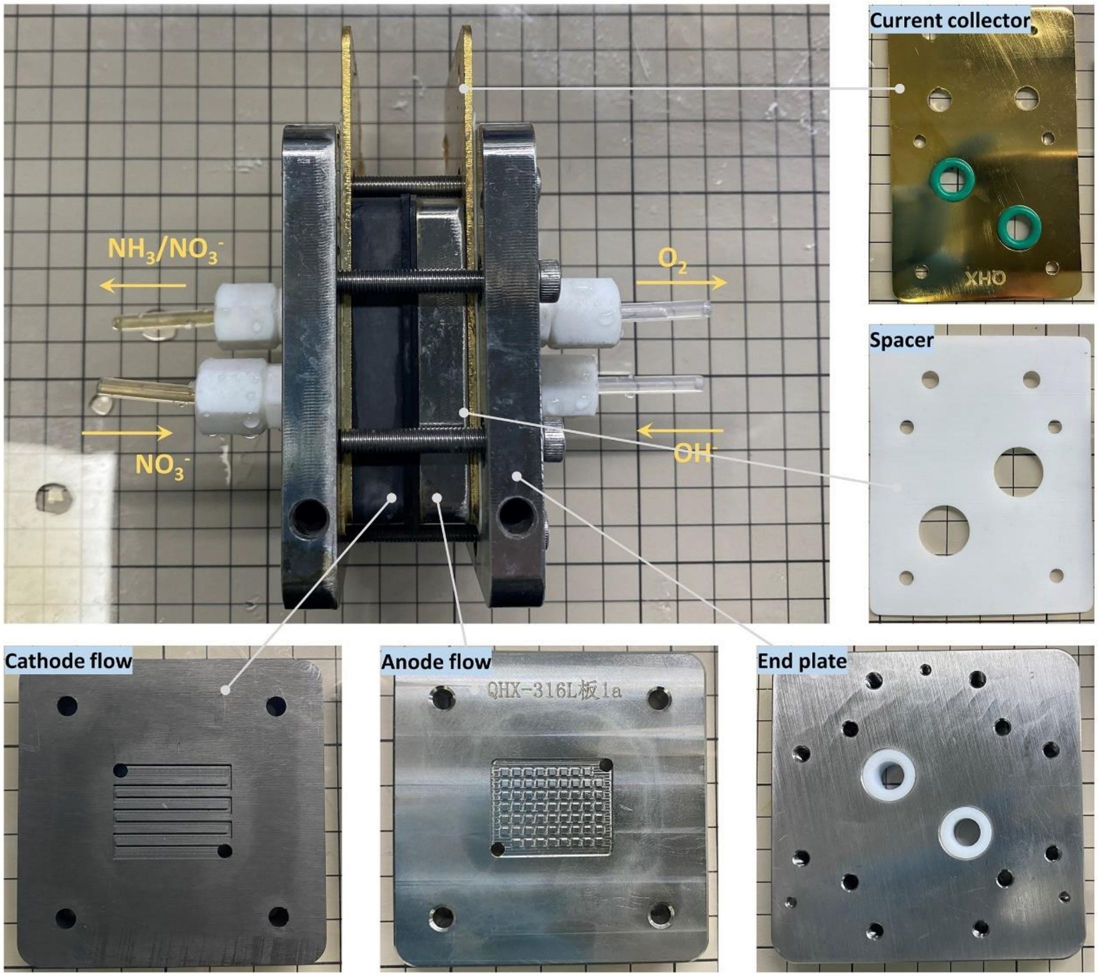

Supplementary Fig. 28 | Photography description of continuous NH_3 electrosynthesis flow cell internal components. Several main parts are included: two current collectors (cooper plates overgild), two spacers (Teflon); two end plates (316L steel); anode flow (316L steel) and cathode flow (graphite).

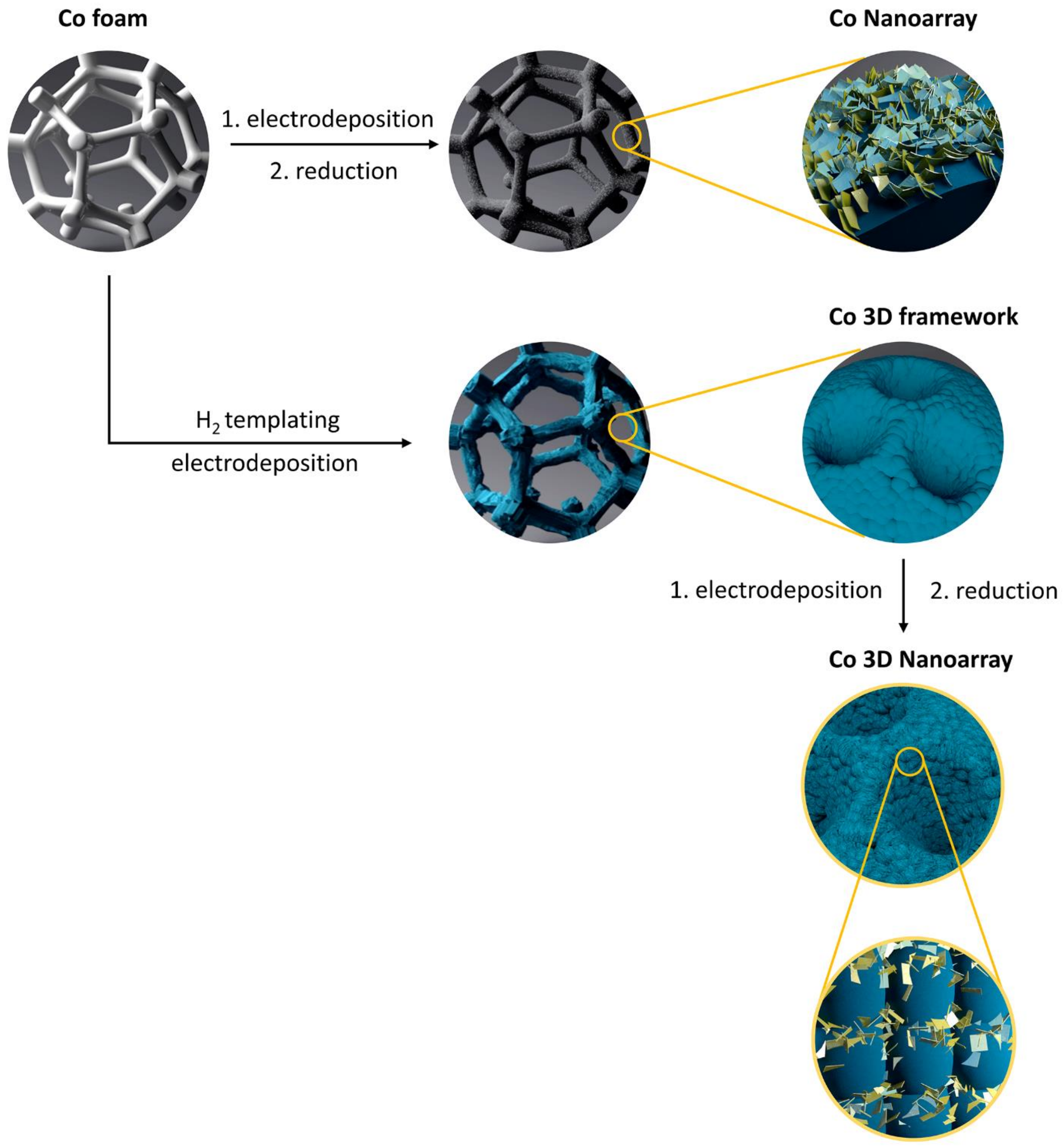

205

206 **Supplementary Fig. 29 |** Schematic illustration of fabricating 1) Co Nanoarray, 2) Co 3D framework and

207 3) Co 3D Nanoarray as NO_3^-RR catalyst.

208

209

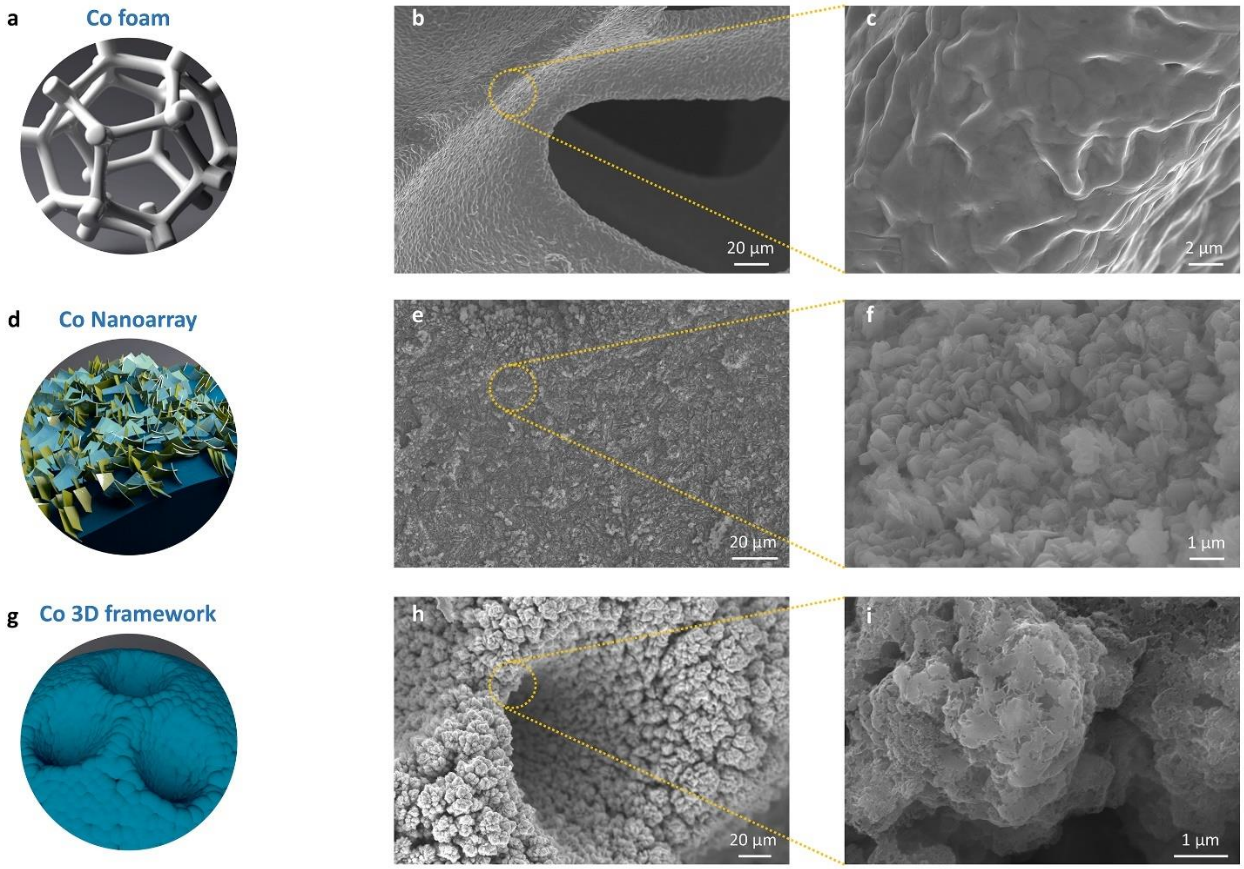

Supplementary Fig. 30 | Microscopic SEM images of referred NO_3^-RR catalyst: **a,b,c,** Co foam; **d,e,f,** Co Nanoarray; **g,h,i,** Co 3D framework.

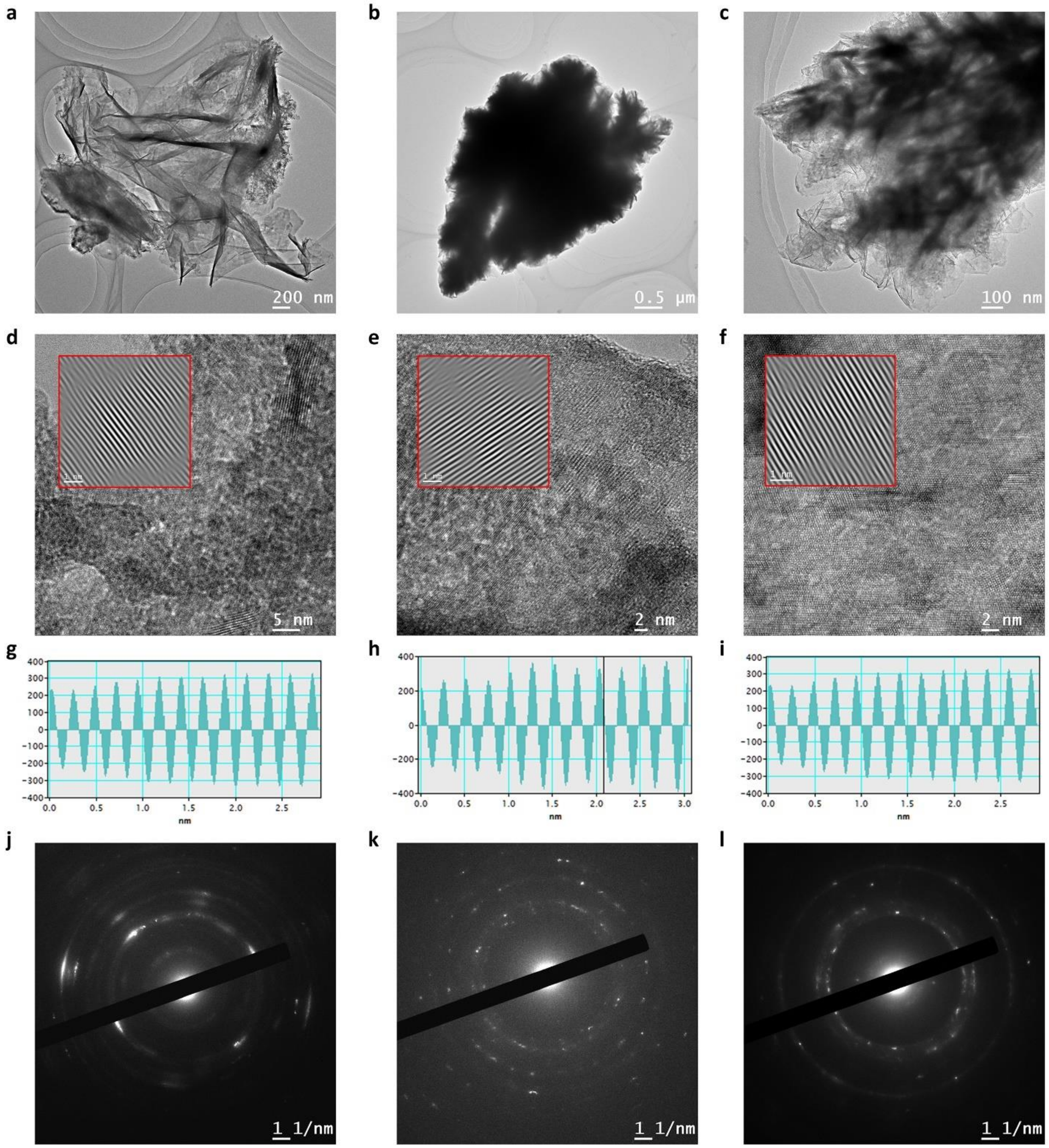

Supplementary Fig. 31 | TEM images of synthesized Co catalysts particles obtained via ultra-sonification from substrate: **a,** Co nanoarray; **b,** Co 3D framework; **c,** Co 3D Nanoarray. **d,e,f,** and **g,h,i,** show high resolution images and lattice spacing image of the particles, respectively. **j,k,l,** shows SAED pattern of synthesized catalysts, respectively.

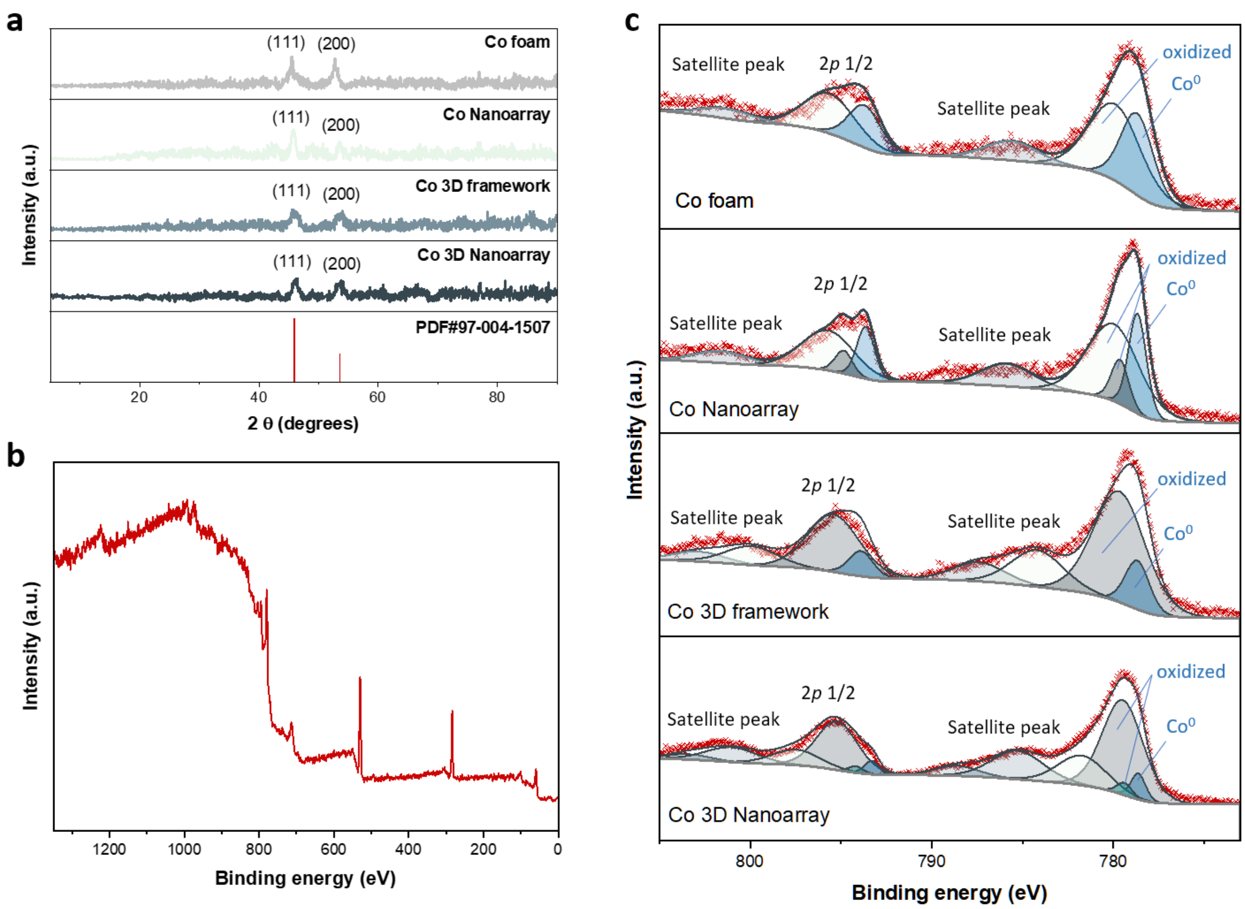

Supplementary Fig. 32 | a, Powder XRD data of synthesized Co foam, Co Nanoarray, Co 3D framework and Co 3D Nanoarray. **b,** XPS survey of Co 3D Nanoarray. **c,** Co 2*p* XPS data of synthesized Co catalysts with different morphology.

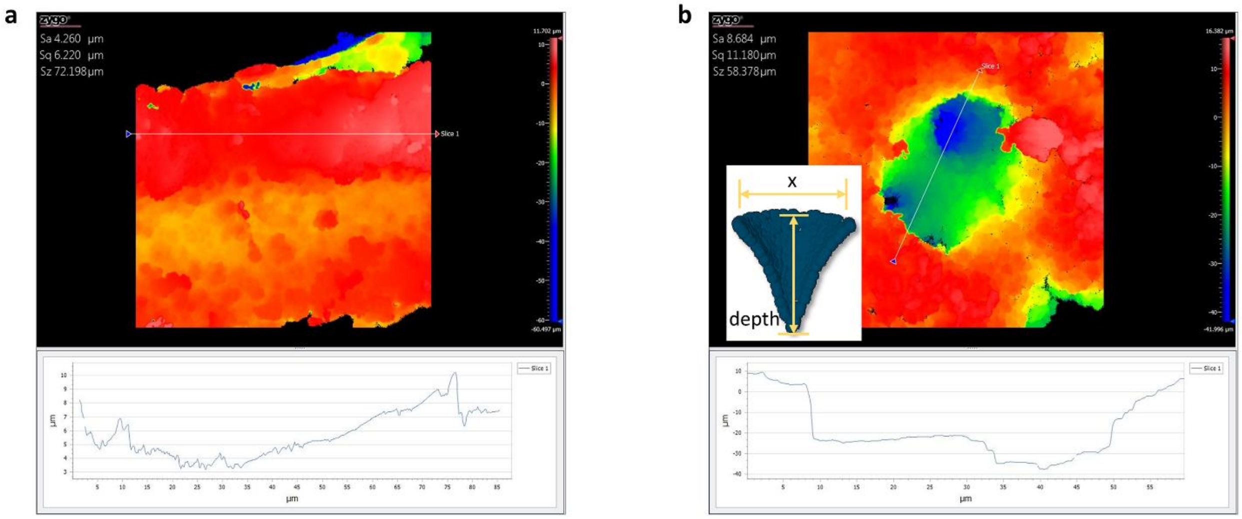

Supplementary Fig. 33 | Surface topography of **a,** Co Nanoarray and **b,** Co 3D Nanoarray obtained by three-dimensional white light interference detection. The lower profile plots the depth versus one-dimensional position of both nanostructures.

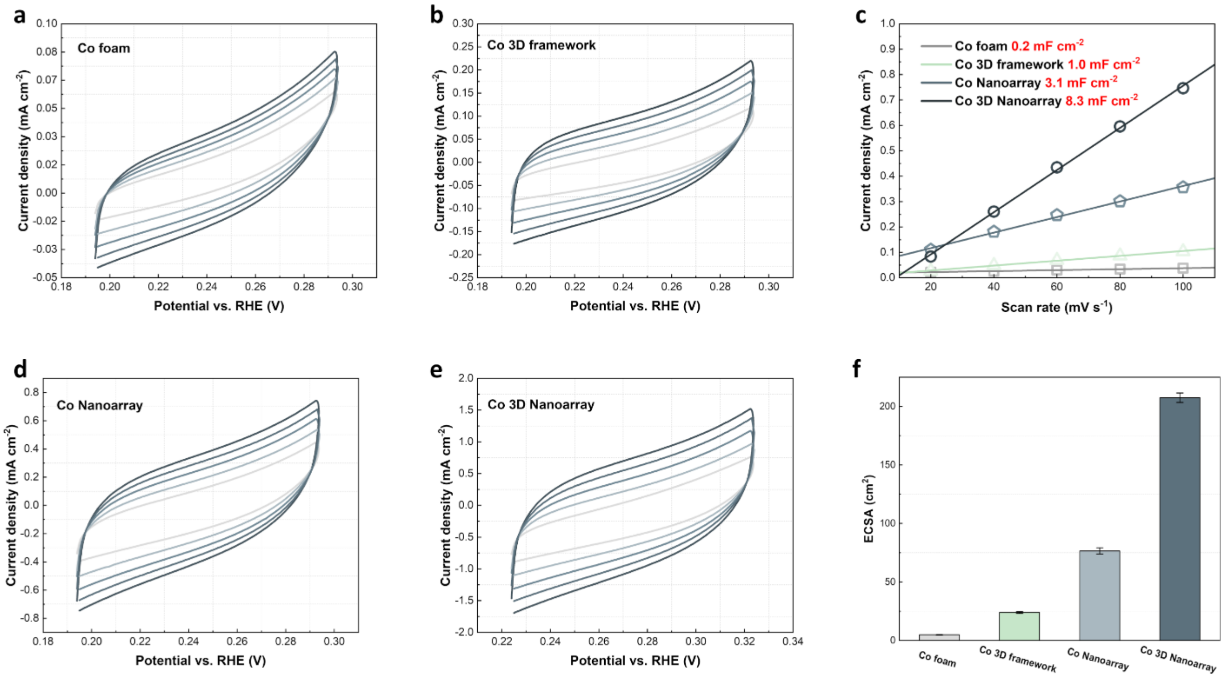

Supplementary Fig. 34 | CV curves of **a,** Co foam, **b,** Co 3D framework, **d,** Co Nanoarray and **e,** Co 3D Nanoarray at 20-100 mV s^{-1}. **c,** and **f,** shows double layer capacitance and ECSA of synthesized Co catalysts, respective.

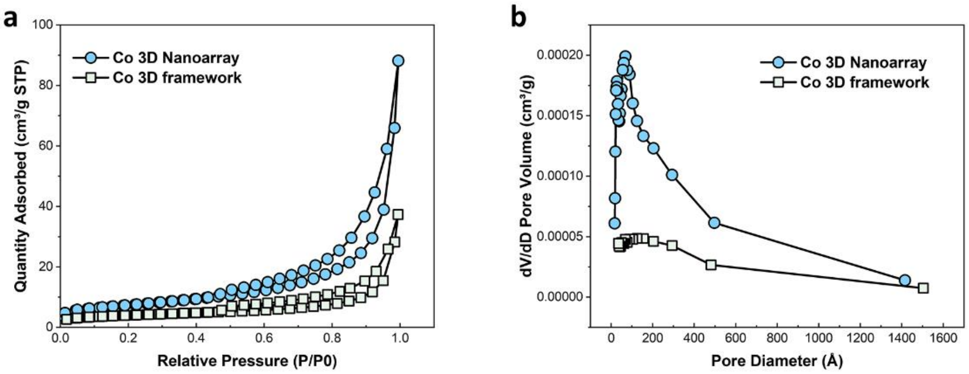

Supplementary Fig. 35 | a, N_2 adsorption-desorption curves of synthesized Co catalysts. **b,** porous size distribution of synthesized Co catalysts.

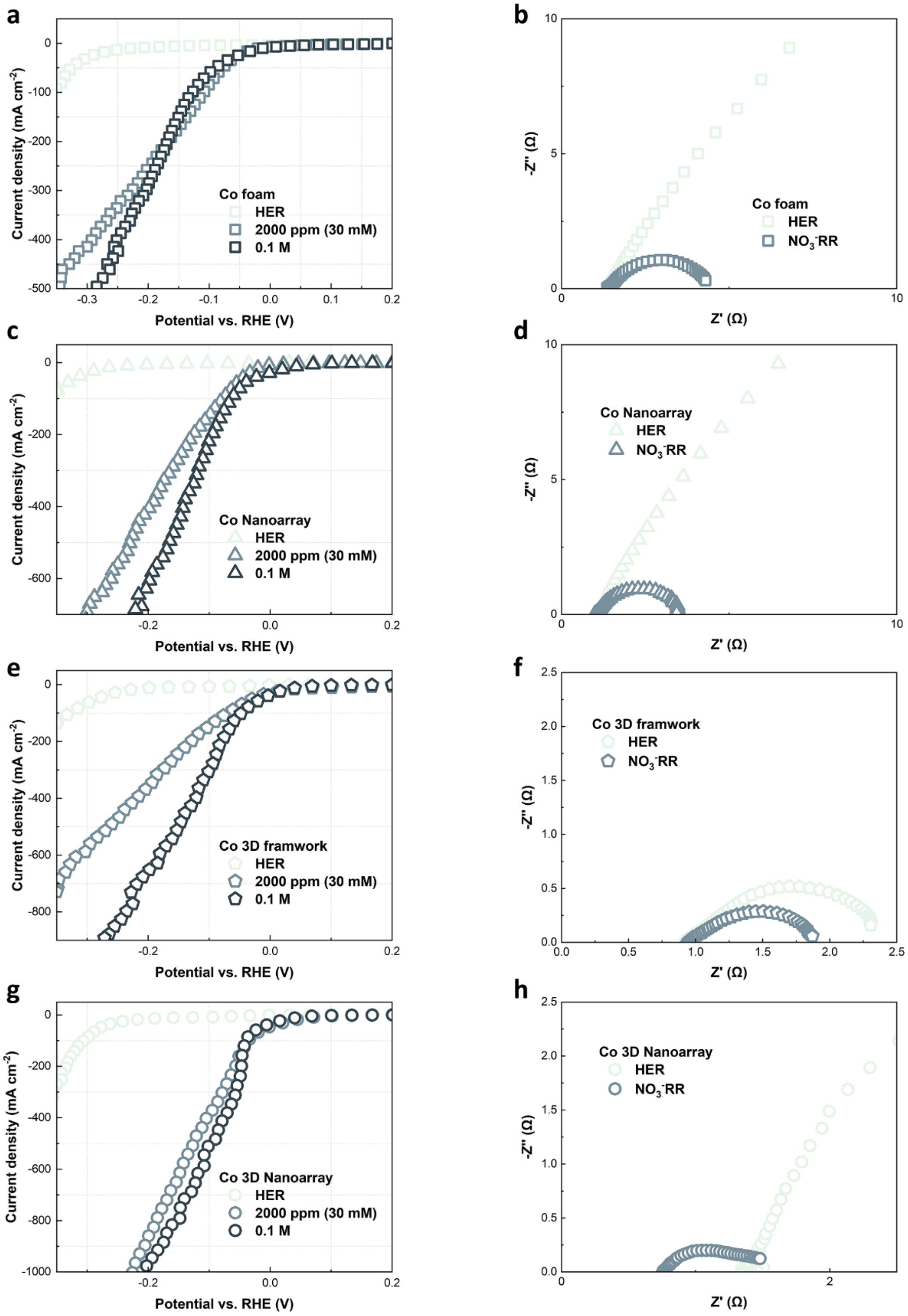

Supplementary Fig. 36 | Linear sweep voltammetry curves and EIS measurements of **a,b,** Co foam; **c,d,** Co Nanoarray; **e,f,** Co 3D framework; **g,h,** Co 3D Nanoarray. The measurements were conducted with 1 M KOH with none (HER), 2000 ppm KNO_3 or 0.1 M KNO_3 (for LSV only), respectively.

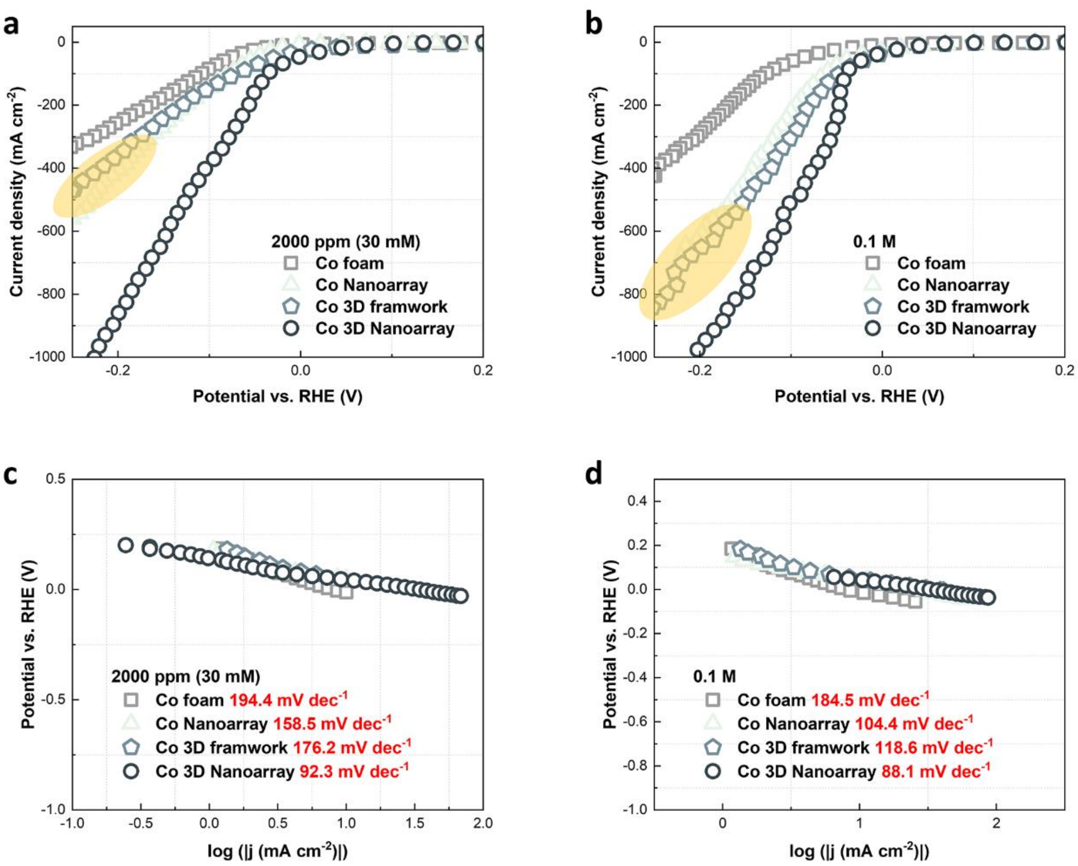

Supplementary Fig. 37 | Comparison of I-V relationships for Co catalysts with different morphologies: **a,** 2000 ppm KNO_3; **b,** 0.1 M KNO_3. **c,** and **d,** show the Tafel plots, respectively.

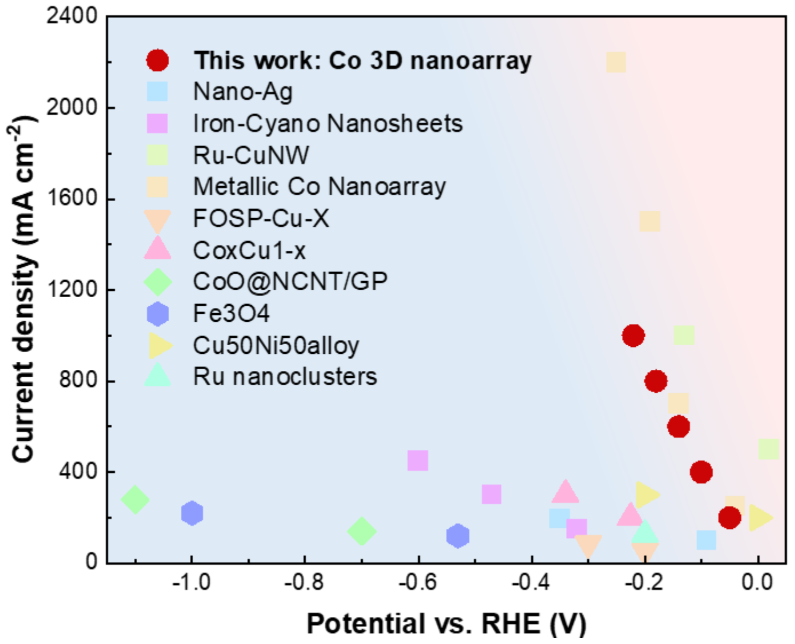

Supplementary Fig. 38 | Comparison data of I-V relationships of NO_3^-RR catalysts that can offer current > 100 mA cm^{-2} recently reported: current density versus potential (RHE).

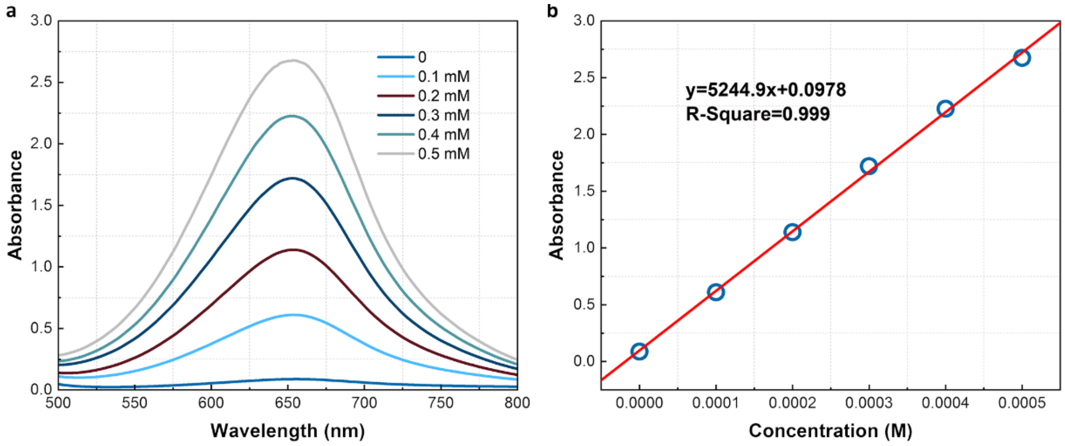

Supplementary Fig. 39 | a, UV-vis curve of 0, 0.1 mM, 0.2 mM, 0.3 mM, 0.4 mM, 0.5 mM NH_3 in 1 M KOH from 500 nm to 800 nm. **b,** standard calibration line of NH_3 in 1 M KOH.

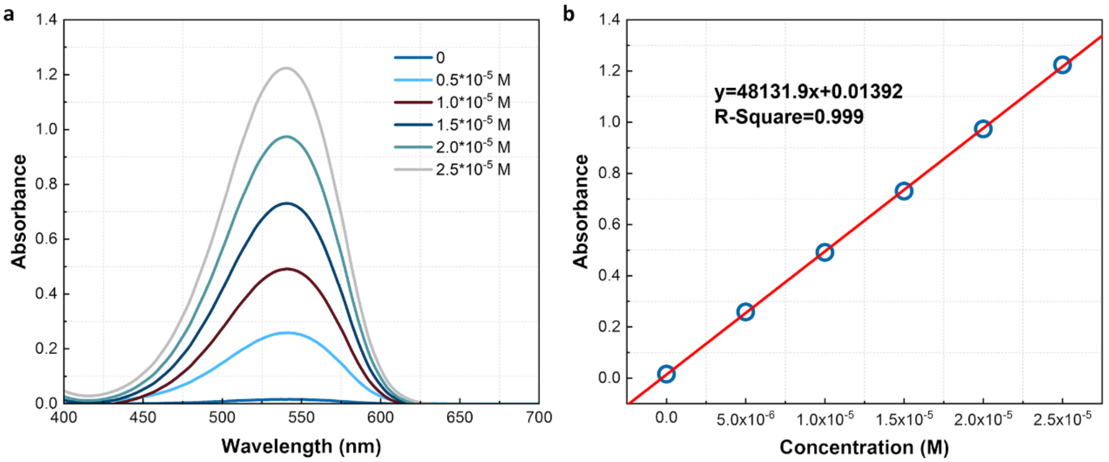

Supplementary Fig. 40 | a, UV-vis curve of 0, $0.5*10^{-5}$ M, $1.0*10^{-5}$ M, $1.5*10^{-5}$ M, $2.0*10^{-5}$ M, $2.5*10^{-5}$ M KNO_2 in neutral pH from 400 to 700nm. **b,** standard calibration line of KNO_2 in neutral pH.

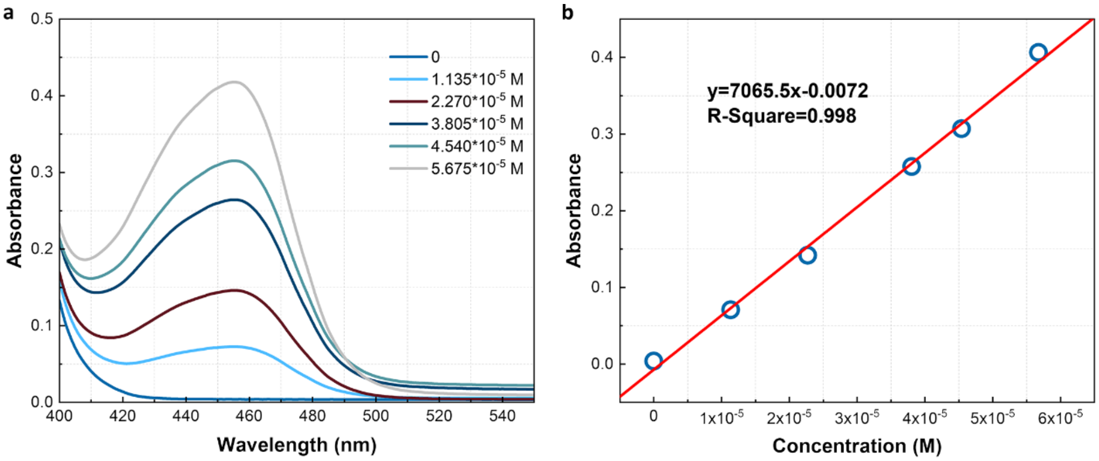

Supplementary Fig. 41 | a, UV-vis curve of 0, $1.135*10^{-5}$ M, $2.270*10^{-5}$ M, $3.805*10^{-5}$bM, $4.540*10^{-5}$ M, $5.675*10^{-5}$ M N_2H_4 in pH=3 from 400 to 550nm. **b,** standard calibration line of N_2H_4 in pH=3.

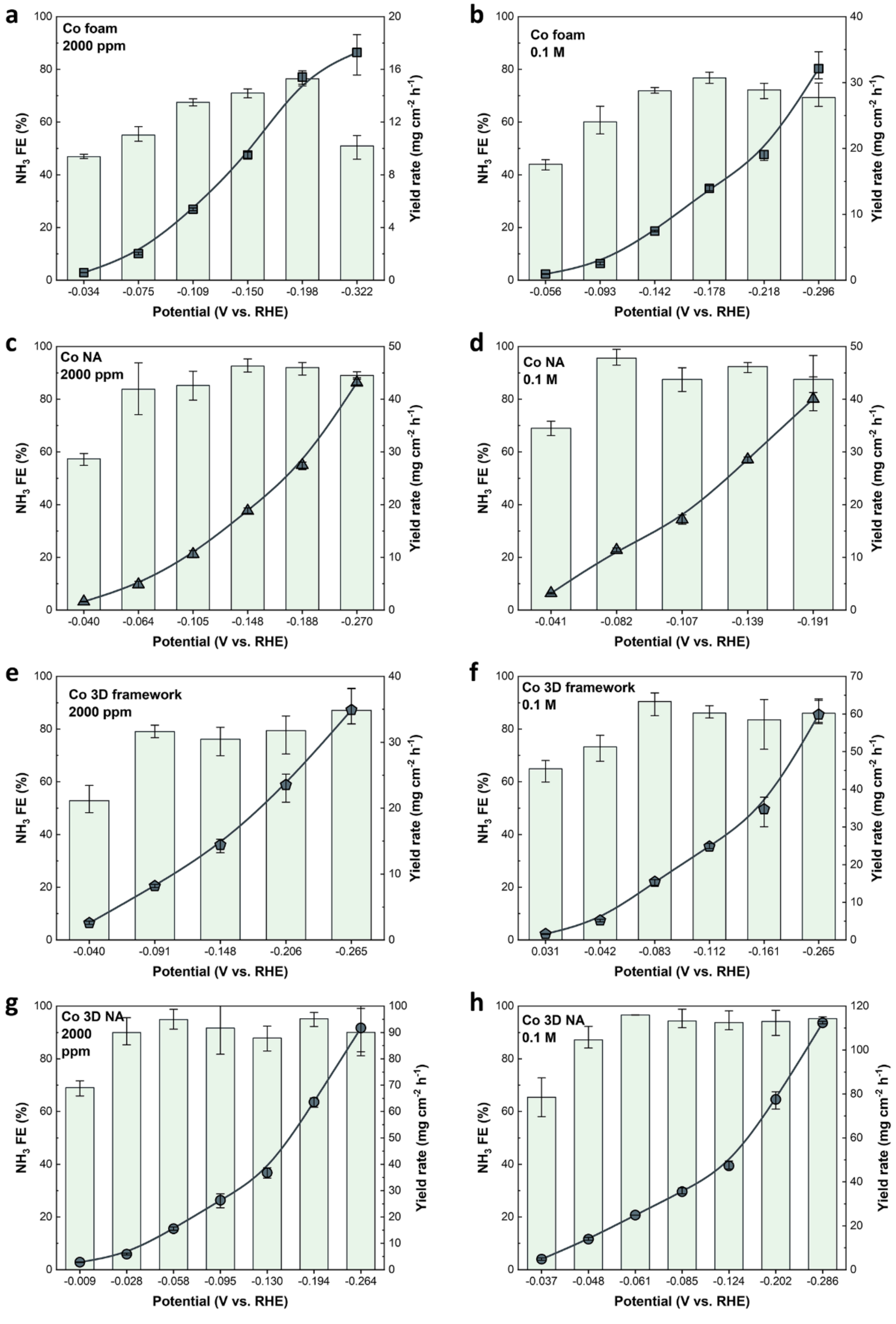

Supplementary Fig. 42 | NH_3 faradaic efficiency (left Y axis) and accordant NH_3 yield rate (right Y axis) of synthesized Co catalysts with 2000 ppm or 0.1 M KNO_3.

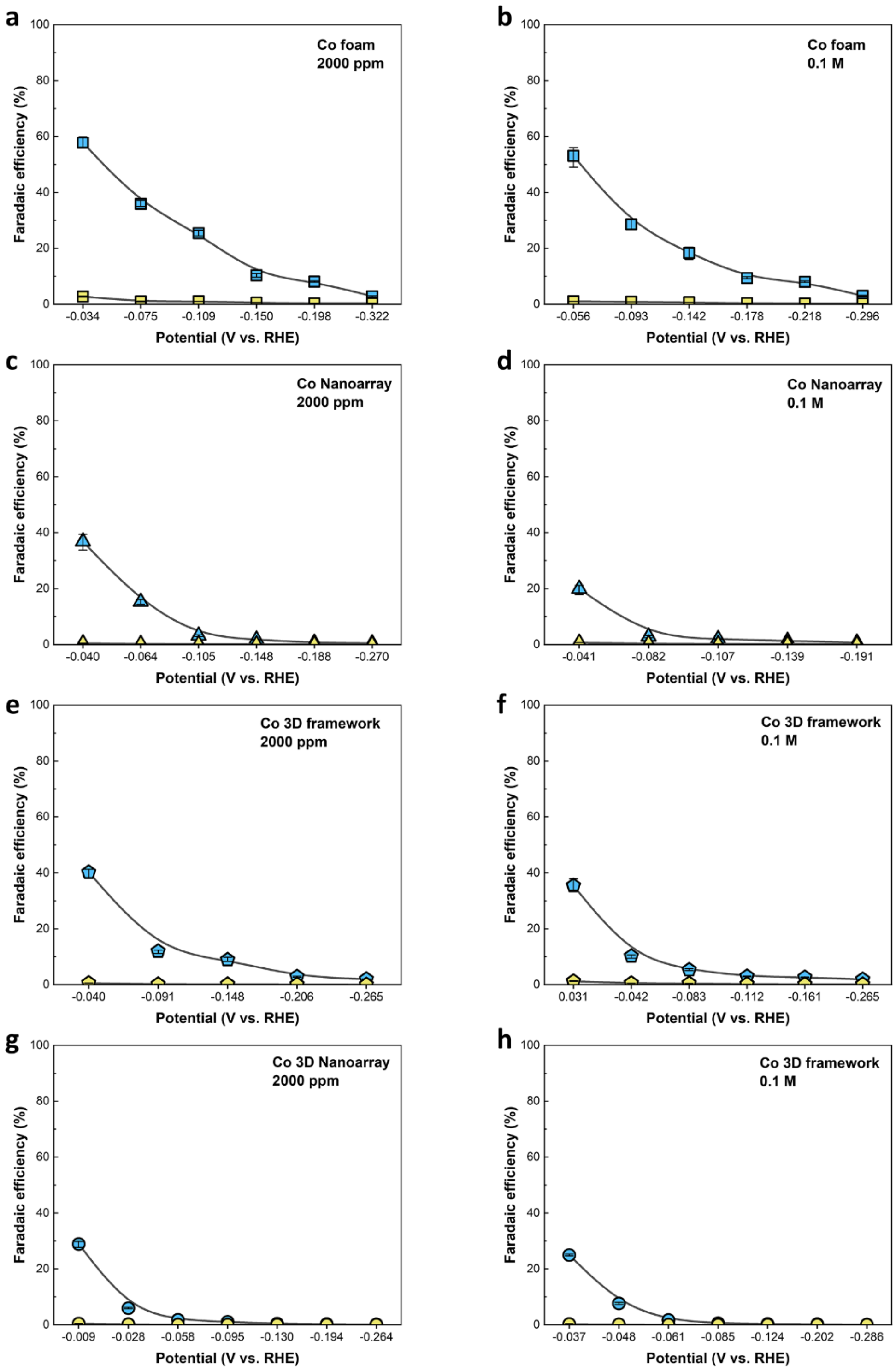

Supplementary Fig. 43 | Side products NO_2^- and N_2H_4 faradaic efficiency synthesized Co catalysts with 2000 ppm or 0.1 M KNO_3. NO_2^- FE are presented in blue symbols and N_2H_4 FE are in yellow ones.

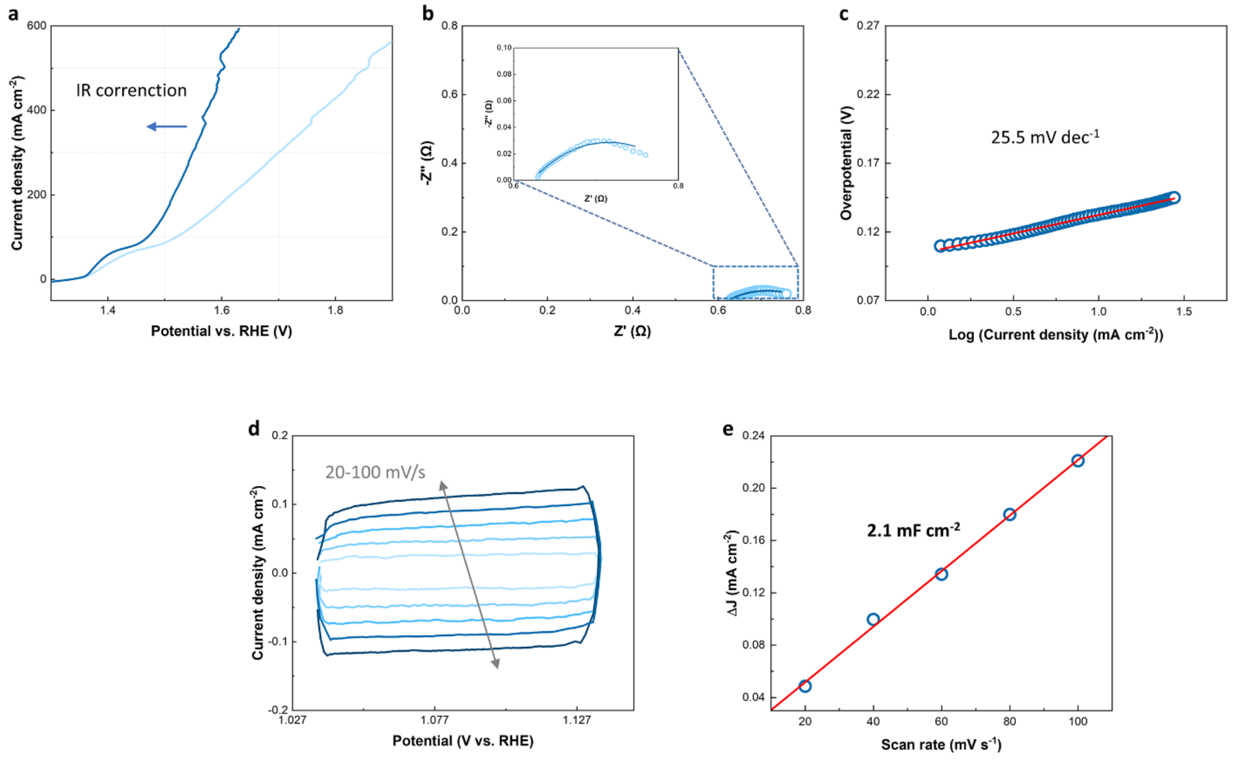

Supplementary Fig. 44 | Electrochemical measurements of NiFe anode for OER in 1M KOH. **a,** polarization curve at a scan rate of 5 mV s^{-1}. **b,** EIS measurements at an oxygen evolution current density of ~10 mA cm^{-2}. **c,** Tafel plot of NiFe for OER. **d,** Cyclic voltammetry of non-faradaic region of NiFe at scan rates 20-100 mV s^{-1} **e,** Plots of current density versus the scan rate for various catalysts for ECSA measurements.

$$ECSA_{NiFe} = 2.1\, mF\, cm^{-2} / 40\, \mu F\, cm^2_{ECSA} \ = 52.5\, cm^2_{ECSA}$$

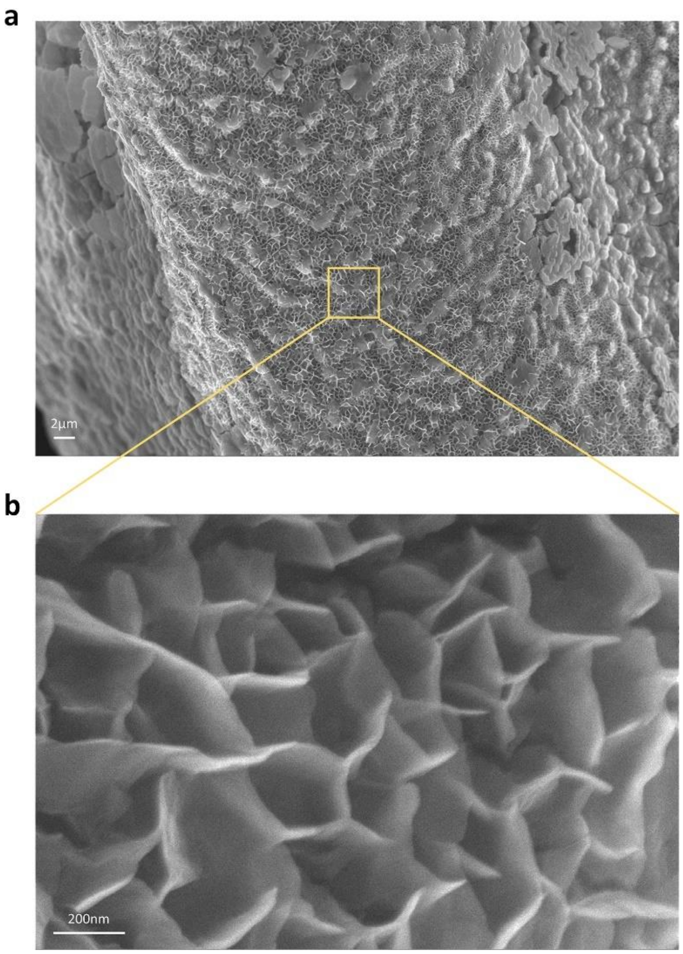

Supplementary Fig. 45 | SEM image of NiFe as anode for OER at different magnification before testing.

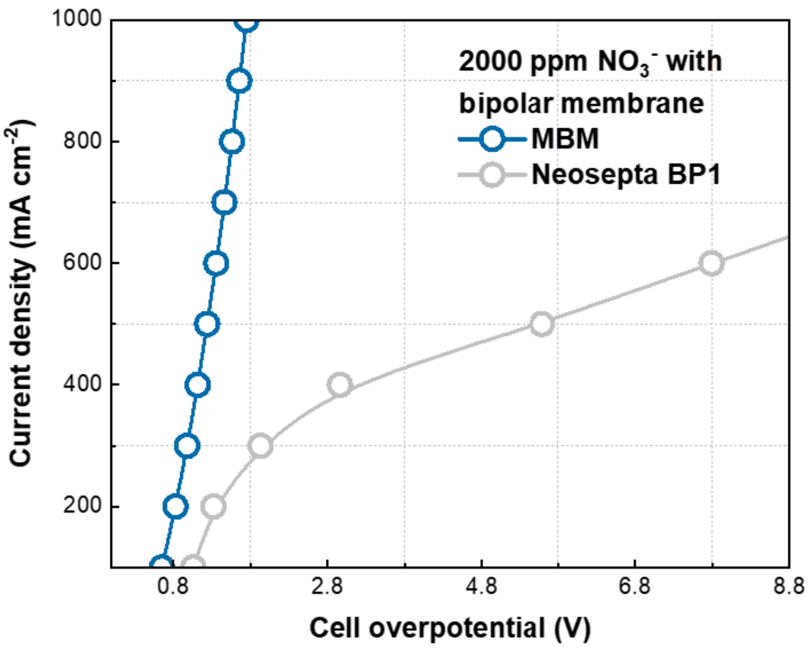

Supplementary Fig. 46 | Relationship between current density and cell overpotential of the bipolar membrane NH_3 electrosynthesis flow cell (2000 ppm NO_3^-).

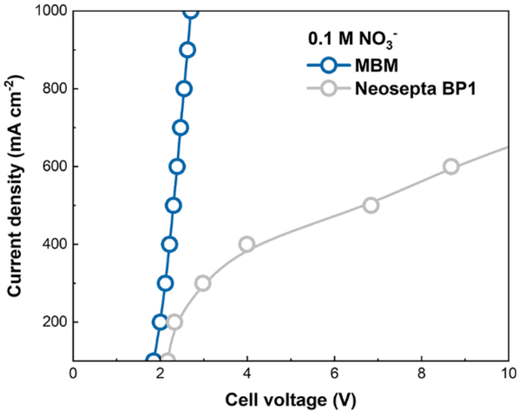

Supplementary Fig. 47 | Polarization curves of electroreduction nitrate to ammonia in aqueous solutions with 1 M KOH/0.1 M KNO_3 in catholyte and 1 M KOH in anolyte.

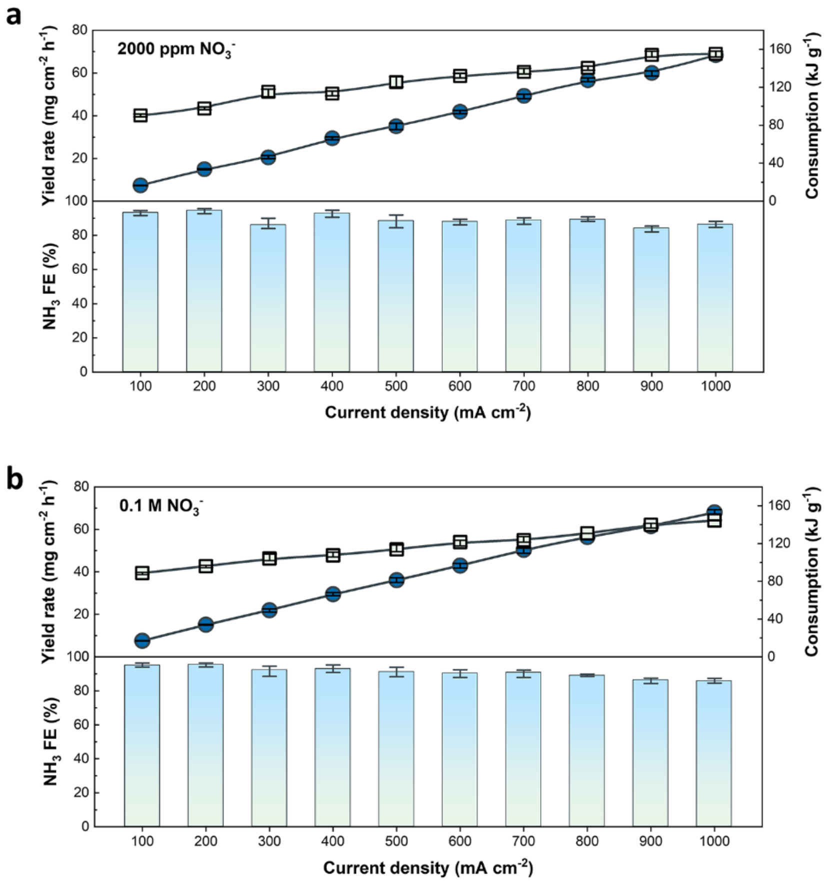

Supplementary Fig. 48 | NH_3 Faradaic efficiencies (lower bars), yield rates (upper circular scatter-line, left Y axis) and energy consumptions (upper rectangular scatter-line, right Y axis) of MBM NH_3 electrosynthesis system with **a,** 2000 ppm and **b,** 0.1 M NO_3^-.

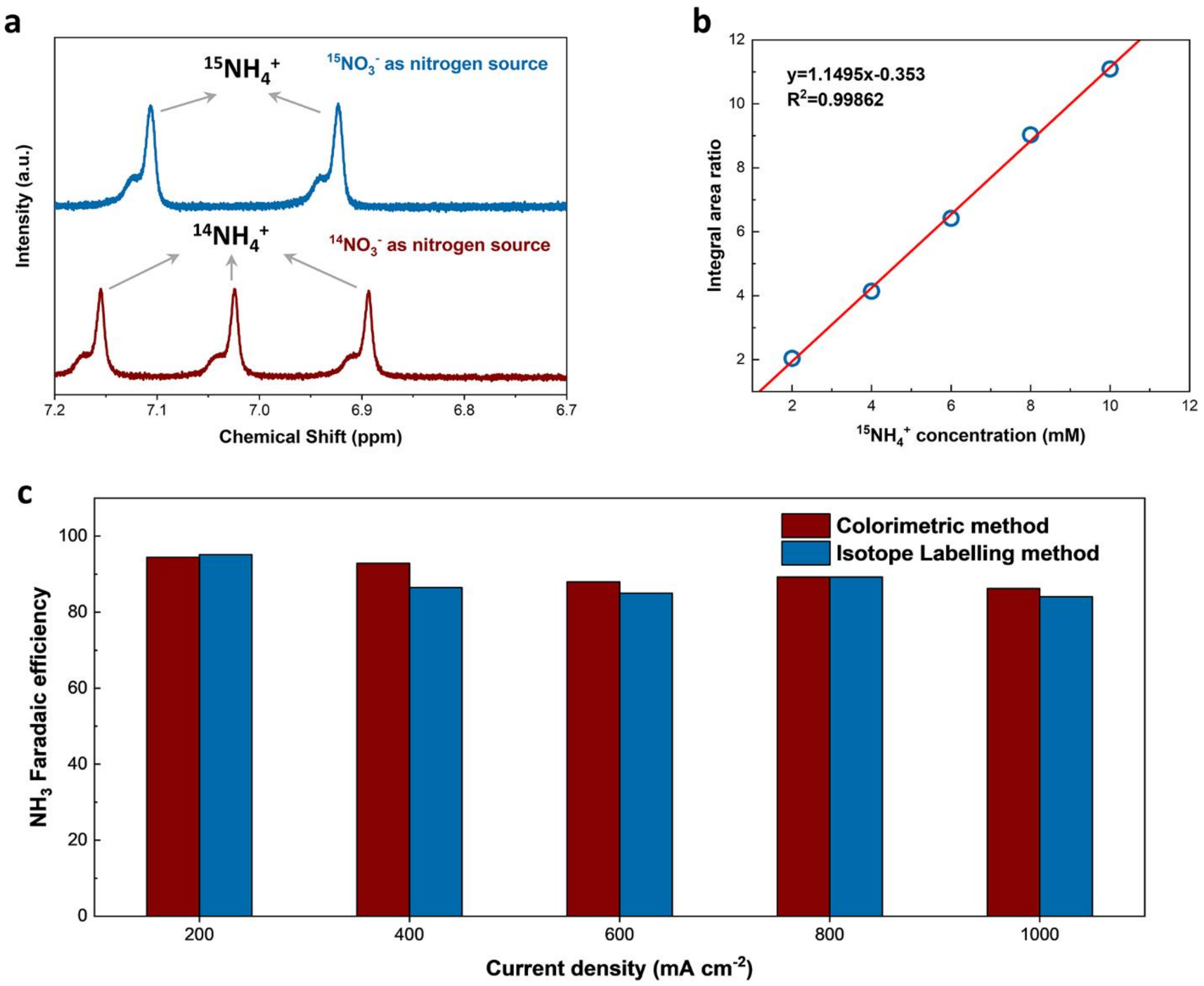

Supplementary Fig. 49 | N-15 isotope labelling experiments for NH_3 determination. **a,** 1H NMR spectrum of products using $^{15}NO_3^-$ and $^{14}NO_3^-$ as N-source. **b,** calibration line of $^{15}NH_4^+$ concentration. **c,** the comparation of Faradaic efficiency between colorimetric method and isotope labelling method.

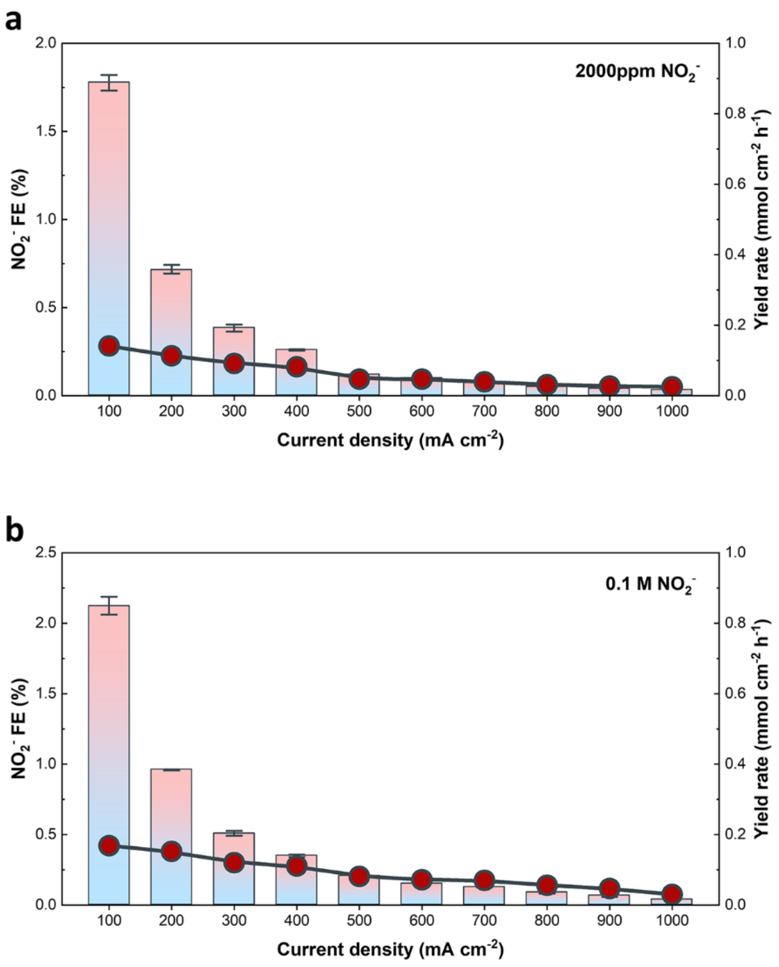

Supplementary Fig. 50 | Side products NO_2^- FE (bars, left Y axis) and yield rates (scatter-line, right Y axis) of MBM NH_3 electrosynthesis systems with **a,** 2000 ppm and **b,** 0.1 M NO_3^-.

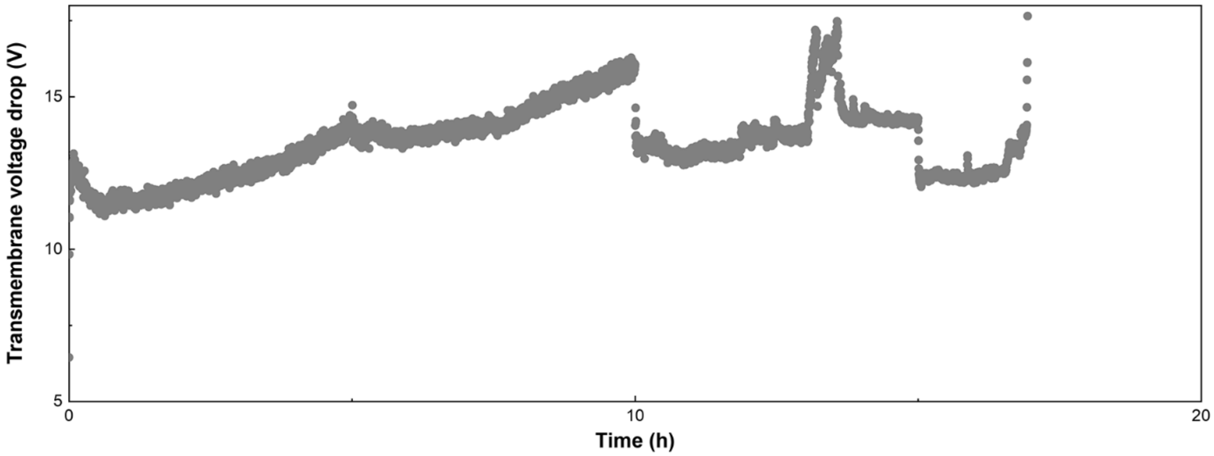

Supplementary Fig. 51 | Stability measurements of NH_3 electrosynthesis system equipped with Commercial BP1 with 1 M OH^- and 1 M NO_3^- at 650 mA cm^{-2}

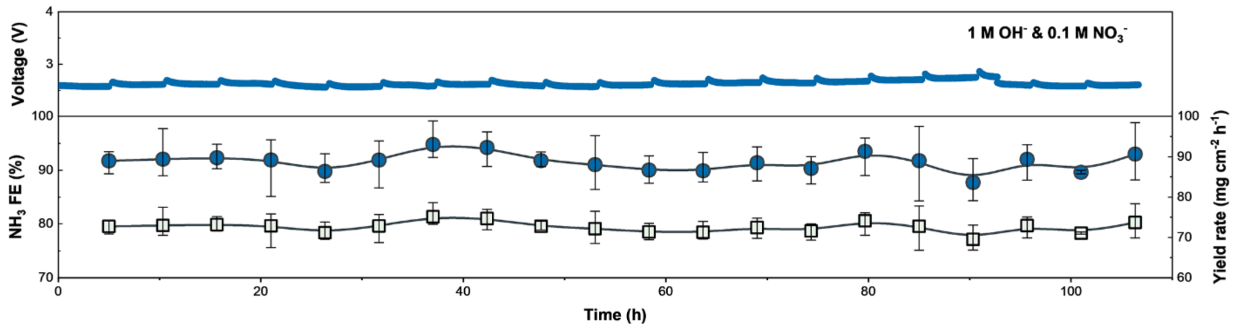

Supplementary Fig. 52 | Stability measurements of MBM NH_3 electrosynthesis systems with 1 M OH^- and 0.1 M NO_3^- at 1 A cm^{-2}. The lower plots show the NH_3 FE (circular scatter-line, left Y axis) and yield rates (rectangular scatter-line, right Y axis) versus time; the upper plot shows the cell voltage change versus time.

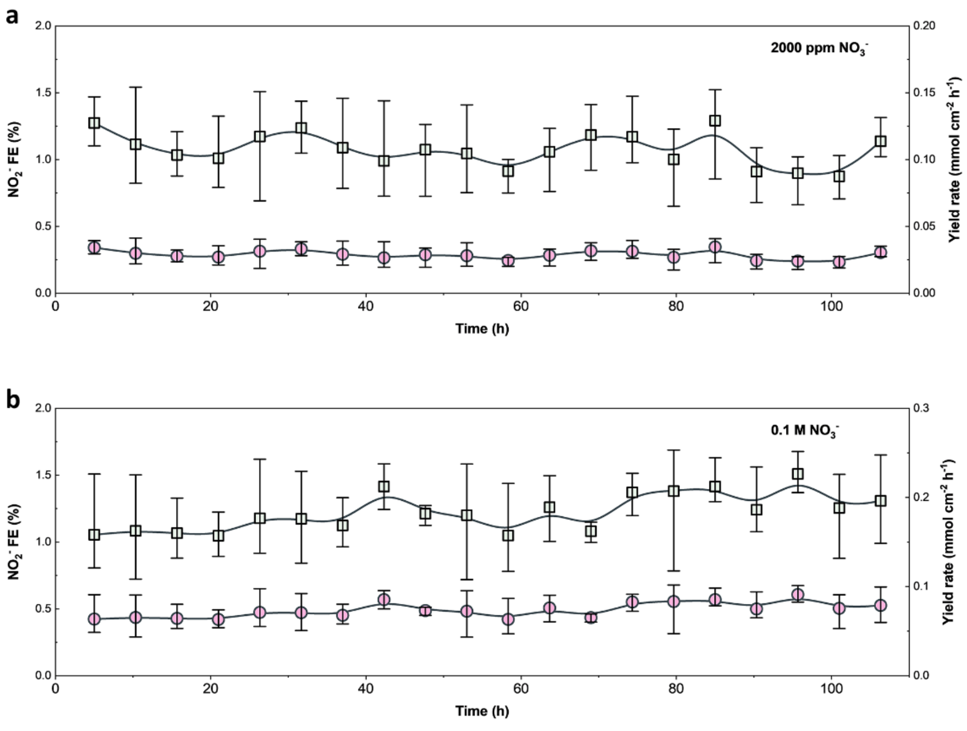

Supplementary Fig. 53 | side products NO_2^- recording during NH_3 synthesis stability measurements with MBM flow cell with **a,** 2000 ppm and **b,** 0.1 M NO_3^-. NO_2^- FE and yield rates are represented by circular (left Y axis) and rectangular (right Y axis), respectively.

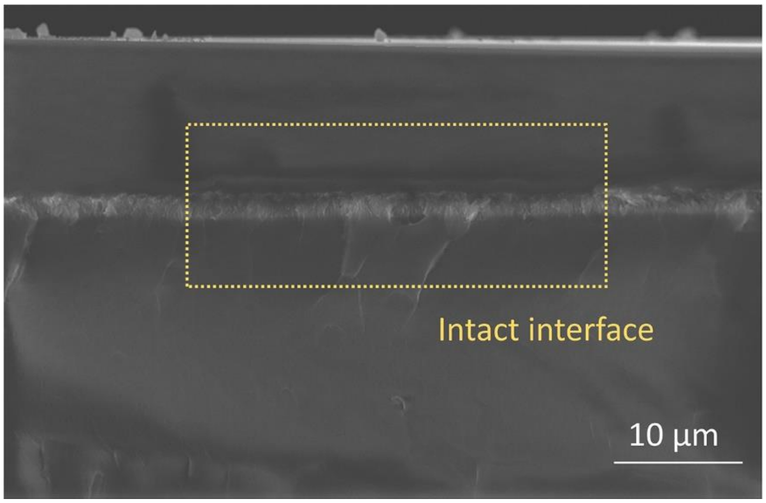

Supplementary Fig. 54 | SEM image of MBM interface morphology after NH_3 electrosynthesis.

II. Supplementary Tables

Supplementary Table 1. The transmembrane voltage drops at 10 mA cm^{-2}, 50 mA cm^{-2} and 100 mA cm^{-2} of bipolar membrane commercial-available or reported in recent 5 years.

**Bipolar membrane**	***U10/V**	**U50/V**	**U100/V**	**Electrolyte(s)**	**year**	**Ref.**
MBM	0.65	0.73	0.76	0.5 M Na_2SO_4	**This work**	
Neosepta BP1	0.68	0.98	1.02	0.5 M Na_2SO_4	Commercial	
SBM-NC2.0	0.8	1.1	-	0.5 M Na_2SO_4	2022	[1]
0.5hFCBM	0.74	0.76	0.77	1 M Na_2SO_4	2022	[2]
HBM-SG10	2.2	4.43	-	0.5 M NaCl	2022	[3]
BPM-E3	1.6	2.6	4	2 M Na_2SO_4	2021	[4]
SCBM	0.8	1.08	1.1	0.5 M NaCl	2021	[5]
SBM-D1.0	0.85	1.5	-	0.5 M NaCl	2021	[6]
PIL-6h-BPM	-	1.4	1.87	1 M Na_2SO_4	2021	[7]
LBL BPM	-	1.3	1.65	1 M $KHNO_3$	2021	[8]
Fe2O3@GO-incorporated BPM	0.6	0.8	0.89	0.5 M NaCl	2020	[9]
Fe(III)@PEI-based BPM	-	1.15	1.8	0.5 M Na_2SO_4	2020	[10]
3D BPM with	-	1.2	1.6	1 M H_2SO_4/1 M	2019	[11]

100 μg cm^{-2} GOx				NaOH		
BPM2	3	6.1	9	2 M NaCl	2019	12
bipolar membrane (I)	-	0.9	1.1	1 M H_2SO_4/1 M NaOH	2018	13
BPMs with MoS2(90)	1.13	2.65	-	0.5 M NaCl	2018	14
BPM-PGO/QGO	2	4.5	-	1 M H_2SO_4/1 M NaOH	2018	15
4GO BPM	1.02	-	-	1 M HCl/1 M NaOH	2018	16
4:1 EDOT/PMA dip-made BPM	1.4	-	-	1 M $NaClO_4$	2017	17
MIL-101 bipolar membrane	1.05	3.6	4.2	0.5 M NaCl	2017	18
BPM-3D	0.8	0.95	1	0.5 M Na_2SO_4	2017	19
BPM-LYS	1.8	3.1	-	0.1 M NaCl	2017	20

* U10, U50, U100 represents for the transmembrane voltage drops at WD current of 10 mA cm^{-2}, 50 mA cm^{-2} and 100 mA cm^{-2}.

Supplementary Table 2. Comparison of NH_3 electrosynthesis systems from nitrate with high performance recently reported.

Electrochemical device	NH_3 Faradaic efficiency/%	*Current density/ $mA\ cm^{-2}$	NH_3 yield rate/ $mg\ h^{-1}\ cm^{-2}$	Catalyst	Ref.
bipolar membrane flow cell	86.2 (2000 ppm NO_3^-)	1000	68.4	Co 3D nanoarray	**This work**
batched H-cell	85.9	5.49 (NH_3)	0.435	Cu-PTCDA	[21]
batched H-cell	95.8	52.5 (NH_3)	7.136	Cu/Cu_2O NWAs	[22]
flow cell	86	60.7 (NH_3)	4.812	Fe single atom catalyst	[23]
batched H-cell	35	4.25 (NH_3)	0.337	Pd	[24]
batched H-cell	100	34.6 (NH_3)	2.75	Fe-PPy SAC	[25]
batched H-cell	85	9.65 (NH_3)	0.765	TiO_2-x	[26]
flow cell	82	22 (NH_3)	1.744	Ti	[27]
batched H-cell	100	251 (NH_3)	19.89	Ru nanoclusters	[28]

flow cell	99.1	90 (NH_3)	7.136	$Cu_{50}Ni_{50}$ alloy	29
batched H-cell	91.5	150	12.263	Fe_3O_4	30
batched H-cell	93.8	125	9.041	CoO@NCNT/GP	31
Electro-reaction tank	100	12.8	2.335	GaInSn	32
batched H-cell	95	176 (NH_3)	-	CoxCu1-x	33
batched H-cell	93.91	-	0.1014	FOSP-Cu-X	34
batched H-cell	96	2200 (I-V test)	176.8	Metallic Co Nanoarray	35
	98	500 (stability)	~34		
flow-system H-cell	93	1000 (I-V test)	76.5	Ru-CuNW	36
	>90	400 (stability)	~29.6		
batched H-cell	90	~450	42.1 mg h^{-1} mg_{cat}^{-2}	Iron-Cyano Nanosheets	37
batched H-cell	89.6	194.31	2.89	Nano-Ag	38

* Current density data noted with NH_3 in bracket represents for the value of NH_3 partial current density.

III. Supplementary Notes

Supplementary Note 1. The necessity of using flow cell equipped with bipolar membrane for NH_3 electrosynthesis from nitrate with alkaline electrolytes.

Most reported works for NH_3 electrosynthesis from nitrate are evaluated in H-cell equipped with an cation exchange membrane (normally are Nafion-based) or anion exchange membrane. It cannot be denied that such system with 3-electrode setup can support a convenient and accurate evaluation for the advanced 8-electron catalysts designed in the research works. However, H-cell with monolayer ion exchange membranes might not realize a continuous NH_3 electrosynthesis in an industrial scale for the two reasons as followed:

i) A flow cell system has been proved reveals predominance in aspect of energy consumption compared with H-cell in several electrochemical applications[39, 40], for the membrane electrode assembly design can to the largest extent lower the distance of electrodes; thus, most part of ohmic resistance from electrolytes can be avoided.

ii) As shown in Fig. 1 in the main text, the existence of membrane can avoid the crossover of produced NH_3 from cathode to anode and be re-oxidized again. Nevertheless, a monolayer membrane cannot maintain the balance of ionic potentials for long-term electrolysis. For example, cations (Na^+ or K^+) will play as charge carriers and move from cathode to anode when systems are equipped with proton exchange membrane. The transported cations cannot be consumed by electro-reactions, and can lead to an unbalanced potential of both sides. Such situation can cause an extra energy consumption and might increase with the operation time. Similarity, anion exchange membranes cannot prevent NO_3^- (as reactant) from leaking and mixing with anolyte. On the contrary, bipolar membrane can force the H^+/OH^- producing from water dissociation as charge carriers in the systems, and a continuous process can be achieved by distilling synthesized NH_3 and refilling HNO_3 to cathode termly. Another potential advantage of using bipolar

membrane is the ability of separating electrolytes with different pH. Sometimes the optimal pH for adopted NO_3^-RR and OER catalysts are in consistent, both of which can be operated in unsymmetrical electrolytes with a bipolar membrane.

Supplementary Notes 2. Numerical simulation modelling and detailed discussion of results.

A one-dimensional bipolar membrane model for numerical simulation was built up, as shown in Supplementary Fig. 1., including a symmetrical diffusion boundary layer of 10 μm thichness, a 50 μm cation exchange layer (CEL) and a 50 μm anion exchange layer (AEL). According to the experiments, there is a distinct difference on IEC of both layers (AEL-2.8 mmol cm^{-3}; CEL-0.9 mmol cm^{-3}). Water dissociation (WD) takes place at the interlayer of bipolar membrane. In order to simplify the modelling, interlayers of bipolar membranes were set as an interrupt interlayer. Two types of bipolar membranes were simulated here, including one normal bipolar membrane and one special bipolar membrane with enhanced WD rate as well as ionic transportation.

Two individual parameters were adjusted to describe the difference of both membranes numerically: i) WD constant rate of special membrane was set one-fold higher than that of normal membrane to describe the enhancement of WD reaction. ii) tortuosity ε was set one-fold higher of special membrane to describe the increase of ionic diffusivity in membrane. For explanation of ii), there is no thickness of interlayer in the simplified 1D model, thus ionic transportation from interlayer to membrane layer can be considered as one part of whole ionic diffusivity process and can influence the value of ε.

The operation was set to perform in 0.5 M Na_2SO_4 in a reverse bias of bipolar membrane, when H^+ and OH^- can be generated at the interfacial region. As previous reported, the potential distribution and ionic transport obey Poisson's equation and the Nernst-Planck equation[41, 42]. Also, WD constant rate can affected by electric field based on Onsager's weak electrolyte theory[43, 44]. All parameters and initial conditions referred for simulations are listed in the table as followed[45]:

Parameter	Value	Description
L_mem	0.05[mm]	Membrane thickness
L_sdl	0.01[mm]	Diffusion boundary
c0	0.5[kmol/m^3]	Initial concentration

delta_phil	0[V]	Initial cell voltage
c_fixCEM	0.9[kmol/m^3]	CEM Membrane fix charge
Kw_0	10.64e-14[M^2]	Water dissociation constant at zero field
T	25[degC]	Temperature
A	0.5[K^2*m/V]	Wien effect coefficient (by Onsager relation)
eps_r	78	permitivity
D_H	9.312e-5[cm^2/s]	Diffusivity
D_OH	5.26e-5[cm^2/s]	Diffusivity
kw_r	5e11[M^-1*s^-1]	Backward rate constant water dissociation
c_fixAEM	2.8[kmol/m^3]	AEM Membrane fix charge
D_Na	1.334e-5[cm^2/s]	Diffusivity
D_SO4	1.065e-5[cm^2/s]	Diffusivity
ε	0.05	electrolyte volume fraction

All equations used for numerical simulations are listed and were programmed and solved with COMSOL v 5.6 (1-6):

$$b = \max\left(A * \frac{\frac{abs(E)}{eps_r}}{T^2}, eps \right) \tag{1}$$

B is Help variable

$$E = -d(phil, x) \tag{2}$$

E is Electric field

$$Kw = Kw_0 * max\left(\frac{besselj(1,(2*sqrt(2*b)*i))}{i*sqrt(max(b,eps))}, 1\right) * sqrt\left(\frac{1}{2}\right) \quad (3),$$

using Bessel function from Onsager relation

$$kw_f = Kw * kw_r \quad (4)$$

kw_f is Forward rate constant

$$rw = kw_f - kw_r * max(cOH, eps^2) * max(cH, eps^2) \quad (5)$$

Reaction rate, water autoprotolysis

$$kappa = {F_{const}}^2 * \frac{cH * D_H + cOH * D_{OH} + cNa * D_{Na} + cSO4 * D_{SO4}}{R_{const} * T} \quad (6)$$

kappa is Conductivity

Detailed discussion of simulation results:

All discussions are based on the understanding to separate kinetics of bipolar membrane interface to two parts: including one WD reaction followed by produced H^+ and OH^- transport to membrane layers (Supplementary Fig. 4b). These two processes can be affirmed more accurately in EIS measurements according to the equivalent circuit applied in this work. As shown in Figure 2a and Supplementary Fig. 2, the concentrations of H^+ and OH^- can be obviously increased at the same transmembrane voltage drop after lifting the WD constant rate and tortuosity. Such results keep valid from 0.6V to 1.3V. To be mentioned here plenty of previous work mentioned a thermodynamic equilibrium potential for WD is 0.83V though, this only come into force when the pH difference (ΔpH=14) as presented in the following equation (7)[46]:

$$E^0_{junction} = \frac{RT}{F} ln(\frac{a^{H^+}_{CEM}}{a^{H^+}_{AEM}}) \quad (7)$$

The concentration enhancement indicates that kinetics of WD reaction and ionic diffusivity can influence the overall WD rate of bipolar membrane interface, and this effect becomes valid in quite a wide range of voltage. However, such influence become lower by degrees when transmembrane voltage drop become relative higher. This might be ascribed to that both WD constant rate and ionic transportation can be enhanced versus voltage, and the difference of original value become more inconspicuous. In conclusion, the enhancement of overall kinetics shows most significance and acts as rate-limiting step in a specific voltage range (or current density range).

On the other hand, concentration distribution of Na^+ and SO_4^{2-} of both kind of bipolar membranes shows similarity (Supplementary Fig. 3). This phenomenon can be explanation by “inert” ions that only decided by Poisson’s equation and the Nernst-Planck equation, and IEC as well as diffusivity of inert ions are kept same.

As shown in Supplementary Fig. 4, the consequential I-V relationships of kinetics enhanced bipolar shows preponderance compared to the normal one. Enhancing one of the kinetics factors (no matter WD constant rate or ionic diffusivity) can partially promote the bipolar membrane water dissociation current density. Combining the results from Supplementary Fig. 3, this improvement can confidently ascribe to the produce of H^+/OH^-.

Supplementary Note 3. Data statistics method of CoNi array height value frequency as templates grow on Ni substrate.

The three-dimensional lattice coordination of surface morphology was obtained by white light interferometry measurements. The statistics logic is based on finding the point which is lower than the 8 points surrounded and defined as “peak”. Moreover, an extra restriction that the distances of two peaks should larger than 100 nm was added for the resolution limitation of the characterization. The peak searching and counting were completed by Python based on the following code:

```
import pandas as pd
import numpy as np

data = pd.read_csv('data.txt', sep=' ', )
x_size = data['x'].max()+1
y_size = data['y'].max()+1
z_np = np.zeros((x_size, y_size))

for _, row in data.iterrows():
    if row['z'] ==  'NA':
        row['z'] = -1e10
    z_np[int(row['x']), int(row['y'])] = row['z']

rela_z = []
for x in range(1, x_size-1):
    for y in range(1, y_size-1):
        comp = [z_np[x-1,y-1], z_np[x-1, y+1], z_np[x-1, y],
                z_np[x+1, y-1], z_np[x+1,y+1], z_np[x+1, y],
                z_np[x, y-1], z_np[x, y+1]]
        if z_np[x,y] >= max(comp):
            flag = True
            for exis in rela_z:
                if (exis[0]-x)**2+(exis[1]-y)**2 <= 100:
```

```
                    flag = False
                    break
            if flag:
                rela_z.append([x, y, z_np[x,y], z_np[x,y]-max(comp)])

df = pd.DataFrame(rela_z, columns = ['x', 'y', 'z', 'rela_z'])
df.to_csv('./kevin_rela.csv')
```

Supplementary Note 4. Discussion of bipolar membrane interfacial kinetics varied by interlayer structure obtained by EIS measurements and the contributions to the overall WD performances.

According to the discussion in the main body, the physical size of interlayer structure can be regulated by the template morphology, thus can make influence on interfacial kinetics of bipolar membranes. Under the reverse bias operation mode, the equivalent circuit was set for describing bipolar membranes, composed of series-wound three parts reasonably as several previous reported. Here five parameters obtained from fitted results are mainly discussed here, including ohmic resistance, ionic transportation resistance, capacitance of depletion layer, depletion layer thickness, WD constant. All the EIS experiments were conducted at 5 mA cm^{-2} galvanostatic mode and the acquired kinetic parameters might only take effects at WD current density of small scale[9, 16, 47].

Among these parameters, ohmic resistance mainly resulted by the conductivity of membrane layer and decided by chemical structure and thickness of AEL/CEL, thus received minor impact from variation of bipolar membrane interlayer structure. Ionic transportation resistance in this work was used to describe the difficulty produced H^+ and OH^- transported into membrane layers. In some other reports, such resistance was directly applied for explaining the complexity of WD reaction, which we think as inaccuracy. This resistance is usually obtained at a medium frequency and defined as "charge transfer resistance" at double electrode layer if Faraday process happens, so it should be considered to link a kind of mass transfer process. WD reaction indeed can influence, for it will affect the H^+ and OH^- concentrations, but might not be a decisive factor of ionic transportation resistance. Capacitance of depletion layer is based on the assumption of bipolar membrane as a double electric layer capacitor, and a higher capacitance here can be ascribed to a larger active area at AEL/CEL interface of bipolar membranes. Depletion layer thickness is a parameter that derived from capacitance of depletion layer thickness, which is not independent. Depletion layer thickness is always negative correlated to the capacitance and a thinner depletion layer often foreboded a better transportation behavior of bipolar membrane interlayer; however, the above mentioned conclusions sometimes lose efficacy when the

interlayer structure is far from planes[5], e.g., for MBM(s) in this work. WD constant is obtained from the fitting results of Gerischer element at low frequency region of EIS. This parameter can describe the water dissociation rate (to produce H^+ and OH^-) at interface of bipolar membranes.

As shown in Supplementary Fig. 21, capacitance of depletion layer increases obviously with the lifting of embedded interlayer area, thus, the depletion layer thickness gradually decreased. On the contrary, ionic transportation resistance measured disadvantageously enlarged at certain current density. On the other hand, WD constant shows an optimal value with a medium length of interfacial embedded region, indicating that an excessive increasing of WD sites might go against the catalytic reaction.

Supplementary Fig. 22 shows EIS results of bipolar membranes regulated with width of embedded structure. Different from the above situation, the variation of ionic transportation kinetics is positively correlated, which indicates an optimal structure parameter might existed in the regulated region. Moreover, the enhancement of WD kinetics versus width of embedded interlayer was implying the same WD catalysts might play different roles when physically contact with AEL or CEL, and relative research is ongoing in present.

To be mentioned here, EIS results can only provide one possible explanation based on the specific equivalent circuit, and there is inevitable error occurred in fitting process; however, MBM(s) with whatever interlayer structure parameters measured present enormous advantage over FBM on kinetics, which we believe far beyond the error range of fitting. All fitting results of EIS measurements are summarized in the table below:

	K_d/s^{-1}	Rtr/Ω	n	Q	C/μF cm^2	δ/nm	Rs/Ω
MBM-1	0.50	2.747	0.96	2.68×10^{-5}	1.73×10^{-5}	4.10	3.52
MBM-2	10.64	5.279	0.97	2.86×10^{-5}	2.13×10^{-5}	3.33	1.44
MBM-3	1.87	8.267	0.92	1.27×10^{-4}	7.0×10^{-5}	1.01	1.65
MBM-4	0.31	13.6	0.96	5.27×10^{-6}	3.57×10^{-5}	19.85	1.89

MBM-5	29.92	8.107	0.99	8.12×10^{-6}	7.22×10^{-6}	9.81	2.04
FBM	1.587	107.1	0.98	7.77×10^{-7}	6.54×10^{-7}	108.3	3.35

* MBM1-5 are constructed with AEL that transferred from the templates fabricated under the conditions as followed: MBM-1: precursor concentration: 3.2 wt% $CoCl_2\cdot6H_2O$ & 1.6% $NiCl_2\cdot6H_2O$; growth duration: 1.5 hours; MBM-2: precursor concentration: 0.64 wt% $CoCl_2\cdot6H_2O$ & 0.32% $NiCl_2\cdot6H_2O$; growth duration: 3 hours; MBM-3: precursor concentration: 3.2 wt% $CoCl_2\cdot6H_2O$ & 1.6% $NiCl_2\cdot6H_2O$; growth duration: 3 hours; MBM-4: precursor concentration: 6.4 wt% $CoCl_2\cdot6H_2O$ & 3.2% $NiCl_2\cdot6H_2O$; growth duration: 3 hours; MBM-5: precursor concentration: 3.2 wt% $CoCl_2\cdot6H_2O$ & 1.6% $NiCl_2\cdot6H_2O$; growth duration: 4.5 hours.

* Chi-square of fitted results are listed as followed: MBM-1: 0.0061; MBM-2: 0.0091; MBM-3: 0.0104; MBM-4: 0.0074; MBM-5: 0.0195; FBM: 0.0882.

I-V curves can provide evidence of WD performances determined by integrated kinetic factors. Although revealing different WD constant or ionic transportation kinetics, bipolar membranes with various interfacial length (AEL template-transferred with CoNi array of various growth duration) shows similar WD ability at any tested range (Supplementary Fig. 23, 25). On the other hand, bipolar membranes constructed with AEL template-transferred from array synthesized with to low or high precursor concentration represent inferior position of WD performances. Such property of bipolar membrane can be considered contributed synthetically by WD reactions, ionic transportations and membrane conductions. Besides, all evaluated membranes enjoys similar ionic selectivity (Supplementary Fig. 24, 1st limiting current density) for the same membrane layer they adopted.

Supplementary Note 5. Design of cathodic catalyst for bipolar membrane electrosynthesis with low NO_3^- and at high current density.

The main goal of designing a catalyst for NO_3^- reduction is to achieve a high yield rate with relatively low NO_3^- substrate concentrations. Previously works have been adequately proved that cobalt elements can offer a satisficing performance, and our experimental data indicated that commercial Co^0 foam can realize a high faradaic efficiency and yield rate with 1M NO_3^-. Therefore, we selected Co as the sole element to construct the cathodic catalyst for the NH_3 reactor.

The detailed fabrication processes are shown in the Supplementary Fig. 29. We firstly electrodeposited a Co framework at a high current on Co foam, during which a large amount of H_2 occurred and bubbling at the cathode, acting as a template for Co deposition. After the rapid process, a loose Co framework with microporous and 3D morphology was formed with the help of gas template. Then we constructed second-level of Co nanosheets on the existed framework via small current electrodeposition-reduction strategy, and a more elaborate structure can be constructed. This self-supported catalyst was noted as Co 3D nanoarray, and a small current electrodeposition was also directly conducted on a bare Co foam for comparison (note as Co nanoarray).

The original intention of us is to extremely enlarge the activity sites exposure growing on a multi-dimensional framework, which we believe can simultaneously enhance the intrinsic activity and mass transfer of NO_3^- reduction (verified below). As shown in Figure 5c and Supplementary Fig. 30, the high-resolution SEM image of Co nanoarray reveals as nanosheets vertically grown on the substrate, while low magnified image showed that these sheets covered on the flat surface. On the contrary to Co nanoarray, the Co 3D framework shows a loose structure heaped up with Co sphere, and porous can be find with a diameter of several micrometers; however, finer structure can hardly be found in sub micro size. The constructed Co 3D nanoarray combine the structure of both and formed a multi-layer structure, by which means NO_3^- can be more easily transferred through the Co framework to Co nanosheets and low concentration of reactant can be better utilized. In the end, the intrinsic activity can be promised by the

elaborate nanoarray, and meanwhile the mass transfer can be enhanced by the 3D structure of the framework.

On the other hand, the morphology of catalytic particles can tell the same story. As shown in Supplementary Fig. 31, thin nanosheets of Co can be constructed via small current electrodeposition, and the particle fell off from 3D framework shows low transparency under TEM. Constructed by multi-layer structure, the fragments from Co 3D nanoarray reveals nanoarrays inlaying at the edge of larger Co particles, whose active sites can be kindly exposed. The image of HRTEM shows clear lattice fringe for any morphology constructed, and the accordant spacing is 0.24 nm, denoting the oxide of Co (CoO). Same results can also be demonstrated by SAED pattern of synthesized catalysts. Even possessing totally different morphology, the chemical structure of each catalyst shows as the same Co metal under XRD and XPS spectrum (Supplementary Fig. 32). Note that the various valence state of Co detected by XPS can be ascribed to the minor surface-oxidation, which can hardly be detected by XRD.

Three-dimensional white light interference was further conducted to quantitatively describe the morphology difference between Co nanoarray and Co 3D nanoarray. As shown in Supplementary Fig. 33, the roughness of Co nanoarray was around 5 micrometers, while 3D framework offers the catalyst with a depth larger than 40 micrometers. Owing to the unique 3D structure of the catalyst, Co 3D nanoarray can represent a much higher double layer capacitance and ECSA compared to others as shown in Supplementary Fig. 34. The increase of catalytic surface area can also be verified by the N_2 adsorption measurements (Supplementary Fig. 35), which shows that nearly 2 times of surface area can be obtained by growing of thin nanosheets of the framework. It can be speculated that the advantage of multilayer structure of the Co 3D nanoarray is to help the NO_3^- approach to the active sites with the enlarged spatial geometry configuration, and NH_3 synthesis can be proceeded at a high current and lower concentrations.

I-V curves of all Co- based catalysts were detected with 1M KOH alkaline electrolytes with and without (HER) NO_3^- from 2000 ppm to 0.1 M, which is a simulation of industrial effluents. As shown in Supplementary Fig. 36, all the catalyst reveals an obviously higher NO_3^- reduction activity compared to

HER with either electrolyte. Among them, most catalyst reveals a higher electrolytic current at the same potential with higher substrate (0.1 M KNO_3^-), including Co foam & Co Nanoarray & Co 3D framework, but only Co 3D nanoarray with multilayer design shows a similar performance from 2000 ppm to 0.1 M. This phenomenon confirmed the former speculation that Co 3D nanoarray can intensify the NO_3^- transfer process with more reactive sites to achieve a better utilization of reactant in lower concentration, in which situation concentration is less significant as a driving force. The EIS measurements showed that the NO_3^- activity of all Co catalysts far surpassed HER activity in no matter what NO3- concentrations adopted via the smaller R_{ct}, and ohmic resistances are similar to each other for the same metal used. By comparing the I-V curves of four catalysts referred, we can find that commercial Co foam can only offered the smallest electrolytic current at the same potential with 2000 ppm or 0.1 M NO_3^-, and Co 3D nanoarray act as the best one (Supplementary Fig. 37). Interestingly, the Tafel plots of shows that intrinsic reduction activity of each catalyst is close, mainly accounting for the same element Co and similar vale valence state they possess. In detail, the nanoarray of Co revealed small advantage compared to the ones flat or with spherical morphology, which is supposed contributed by the edges of layered structure.

On the consideration of selectivity, a major reaction can happen at cathode as mentioned in main text (8):

$$NO_3{}^- + 8e^- + 6H_2O \rightarrow NH_3 + 9OH^- \quad (8)$$

Which is accompanied with several possible side reactions (9-10):

$$NO_3{}^- + 2e^- + H_2O \rightarrow 2NO_2{}^- + 2OH^- \quad (9)$$

$$2NO_3{}^- + 14e^- + 10H_2O \rightarrow N_2H_4 + 16OH^- \quad (10)$$

The colorimetric method was adopted for determine the selectivity of all catalysts, and the calibration lines for main product NH_3 and side products NO_2^- and N_2H_4 were presented in Supplementary Fig. 38-41. As discussed above, the main goal of us is to select a Co catalyst with high efficiency and yield rate with low concentration of NO_3^-. As shown in Supplementary Fig. 42, Commercial Co foam can only achieve a maximum NH_3 FE of 76.4 % and corresponding yield of 15.4 mg cm^{-2} h^{-1} with 2000 ppm of NO_3^-, and the

yield of NH_3 can be nearly doubled with a higher concentration of NO_3^- adopted to help the transfer process. By induced Co nanoarray, the performance under low concentration situation was effectively promoted, achieving 43.1 mg cm^{-2} h^{-1} of maximum NH_3 yield rate with 2000 ppm NO_3^-. However, the similar high FE can hardly be reproduced by bare Co framework, might accounting for the lack of nanosheets structure. By combining the layered structure in a higher exposed surface way, Co 3D nanoarray can realize a similar FE with a high electrolytic current, so an outstanding yield of 90.0 mg cm^{-2} h^{-1} can be approached with 2000 ppm NO3 substrate. The comparison of FE and yield rate can prove that intrinsic activity and mass transfer offered a synergistic effect to achieve a better performance of NO_3^- reduction to NH_3. On the other side, the side product NO_2^- was measured lower than 2% for Co 3D nanoarray catalysts at relatively lower potentials, and N_2H_4 can be proved negligible (Supplementary Fig. 43), which is ignored in the following experiments.

As a comprehensive consideration, the designed Co 3D nanoarray is qualified as the cathode catalyst for the bipolar membrane NH_3 electrolysis working with high electrolytic current and low NO_3^- concentration.

Supplementary Note 6. Techno-economic analysis of bipolar membrane fabrication and MBM NH_3 electrosynthesis process

i) The calculation of MBM fabrication cost and comparation with commercial bipolar membranes.

Table R1. Chemical prices referred in the bipolar membrane fabrication process. The prices of product can be searched on https://www.alibaba.com.

Name of chemicals	Price (P, \$ kg^{-1})
p-Terphenyl	219.7
1-Methyl-4-piperidone	77.0
Trifluoromethanesulfonic acid	68.6
Trifluoroacetic acid	29.9
Iodomethane	156.7
$CoCl_2 \cdot 6H_2O$	23.3
$NiCl_2 \cdot 6H_2O$	29.6
Ni plate	84.3
PFSA solution	195.5
Dichloromethane, Urea, etc.	18.0
Total	517.5

The cost of chemicals and consumable items for producing MBM can be calculated:

$$COST_{MBM} = \sum Usage\ amount \times P = \$517.5\ m^{-2}$$

According to the data from DONGYUECHEM Co., the total utilities payment takes up 10-15% of consumable items. Here we set 15% as an example:

$$Total\ COST_{MBM} = 150\% \times COST_{MBM} = \$595.1\ m^{-2}$$

Table R2. The costs comparison of MBM and other commercial bipolar membranes. The data can be obtained from fuelcellstore.com or https://www.alibaba.com.

Name	MBM	Fumasep FBM	TRJBM	Xion-BPM*	Neosepta BP1
Price (\$ m^{-2})	595.1	4733.3	2182.8	49671.1	1350

*Only small size is available.The data was calculated from the price of small size membrane

As shown in the Table 2, the cost of MBM represent advantage compared to several commercial bipolar membranes. To be mentioned here, the price of chemical and consumable items taken for calculation here is grabbed as lab-scale price, and might be further decreased in industrial production. Moreover, the PFSA cation exchange layer could also be reconsidered and replaced by other cationic materials with similar function and much lower price, which can obviously squeeze the overall producing cost in further applications.

ii) The techno-economic analysis of bipolar membrane NH_3 electrosynthesis process.

The analysis of techno-economic analysis for NH_3 electrosynthesis is based on the operation of 1 A cm^{-2} and NH_3 FE of 90%. Assuming the flow rate in the electrolysis system as 0.1 mL/min in single pass mode, the daily production was assumed to be 100 ton/day. According to this condition, total current was calculated to be 58.4 MA, and the total area was 5839 m^2 with a total power usage of 174 MW. Based on these parameters, several levelized costs were shown in Table R3.

Table R3. Levelized cost for techno-economic analysis

Name of parameter	Value
Per area cost*	\$3576/m^2
Total cost of flow system	\$20.8 million

BoP cost**	$11.2 million
Electricity cost***	$125280/day
Maintenance cost****	$3728/day

* Per area cost of the stack is estimated for 1 A/cm^2 operation condition. Assuming the instillation factor is 1.2.

** From the DOE analysis, the balance of plant capital cost is 35% of the total cost.

*** The price of electricity was assumed as $0.03 /kWh.

**** The maintenance cost is assumed as 2.5% of capital cost per year.

Assuming the price of product is $1536 /ton, the yearly profit is given as followed:

$$yearly\ profit$$
$$= the\ yearly\ income\ of\ products - yearly\ electricity\ cost - yerly\ maintenance\ cost$$
$$= \$8.9\ million\ /year$$

Assuming an ideal situation for the stack to be maintained, the NH_3 stack is designed for a 5-year of working. The NPV is roughly estimated using the yearly profit value as cash flows per year, and the nominal interest rate is 5%. According to the NPV calculation equation:

$$NPV = \sum_{n=1}^{5} \$8.9\ million \times (\frac{1}{1.05^n}) - \$20.8\ million - \$11.2\ million = \$15.43\ million$$

The NPV of bipolar membrane electrosynthesis could be determined by the project life span, which was revealed as followed:

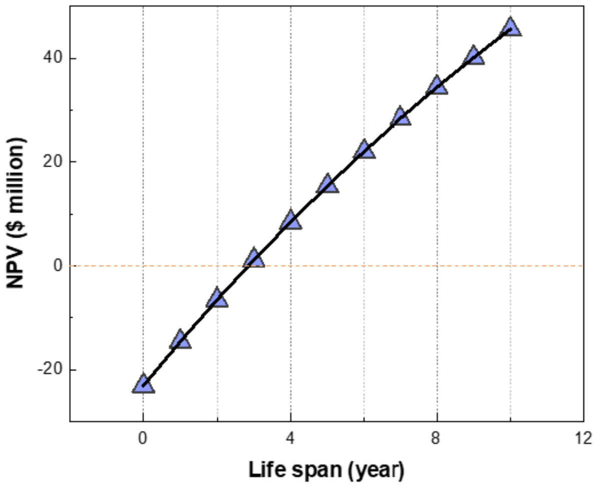

Figure R5. The relationship between calculated NPV and bipolar membrane NH_3 electrosynthesis device life span.

In conclusion, NPV becomes positive when the end-of-life span is around 4 years, which demonstrates this project is a profitable investment. To be mentioned here, most parameters referred here is obtained from lab-scale data, and the cost can definitely be further reduced for industrial-level application. Furthermore, the nitrate reduction is also an eco-friendly technology by solving contamination problem with renewable electricity, which can probably acquire preferential policy in the future. The new technology also brings huge environmental benefits.

IV. Supplementary References

1. Eswaraswamy B, Suhag A, Goel P, Mandal P, Chattopadhyay S. Potential of montmorillonite nanoclay as water dissociation catalyst at the interface of bipolar membrane. *Separation and Purification Technology* **295**, 121257 (2022).

2. Ge Z*, et al.* High-performance bipolar membrane for electrochemical water electrolysis. *Journal of Membrane Science* **656**, 120660 (2022).

3. Eswaraswamy B, Goel P, Mandal P, Chandra A, Chattopadhyay S. Nanocomposite interface coupled with thickness optimization promoting water dissociation in heterogeneous bipolar membrane. *Polymers for Advanced Technologies* **33**, 353-367 (2022).

4. Al-Dhubhani E*, et al.* Entanglement-Enhanced Water Dissociation in Bipolar Membranes with 3D Electrospun Junction and Polymeric Catalyst. *ACS Applied Energy Materials* **4**, 3724-3736 (2021).

5. Shehzad MA*, et al.* Shielded goethite catalyst that enables fast water dissociation in bipolar membranes. *Nature Communications* **12**, 9 (2021).

6. Eswaraswamy B, Mandal P, Goel P, Chattopadhyay S. Potential of Dipicolinic Acid as a Water-Dissociating Catalyst in a Bipolar Membrane. *ACS Applied Polymer Materials* **3**, 6218-6229 (2021).

7. Li G*, et al.* In-situ grown polyaniline catalytic interfacial layer improves water dissociation in bipolar membranes. *Separation and Purification Technology* **275**, 119167 (2021).

8. Yan Z, Hitt JL, Zeng Z, Hickner MA, Mallouk TE. Improving the efficiency of CO2 electrolysis by using a bipolar membrane with a weak-acid cation exchange layer. *Nature Chemistry* **13**, 33-40 (2021).

9. Kim BS*, et al.* Bipolar Membranes to Promote Formation of Tight Ice-Like Water for Efficient and Sustainable Water Splitting. *Small* **16**, 2002641 (2020).

10. Ge Z*, et al.* Beneficial Use of a Coordination Complex As the Junction Catalyst in a Bipolar Membrane. *ACS Applied Energy Materials* **3**, 5765-5773 (2020).

11. Chen Y*, et al.* High-Performance Bipolar Membrane Development for Improved Water Dissociation. *ACS Applied Polymer Materials* **2**, 4559-4569 (2020).

12. Cheng G, Zhao Y, Li W, Zhang J, Wang X, Dong C. Performance enhancement of bipolar membranes modified by Fe complex catalyst. *Journal of Membrane Science* **589**, 117243 (2019).

13. Hohenadel A, Powers D, Wycisk R, Adamski M, Pintauro P, Holdcroft S. Electrochemical Characterization of Hydrocarbon Bipolar Membranes with Varying Junction Morphology. *ACS Applied Energy Materials* **2**, 6817-6824 (2019).

14. Li J, *et al.* Exfoliated MoS2 nanosheets loaded on bipolar exchange membranes interfaces as advanced catalysts for water dissociation. *Separation and Purification Technology* **194**, 416-424 (2018).

15. Manohar M, Das AK, Shahi VK. Efficient Bipolar Membrane with Functionalized Graphene Oxide Interfacial Layer for Water Splitting and Converting Salt into Acid/Base by Electrodialysis. *Industrial & Engineering Chemistry Research* **57**, 1129-1136 (2018).

16. Yan Z, *et al.* The balance of electric field and interfacial catalysis in promoting water dissociation in bipolar membranes. *Energy & Environmental Science* **11**, 2235-2245 (2018).

17. McDonald MB, Freund MS, Hammond PT. Catalytic, Conductive Bipolar Membrane Interfaces through Layer-by-Layer Deposition for the Design of Membrane-Integrated Artificial Photosynthesis Systems. *ChemSusChem* **10**, 4599-4609 (2017).

18. Wang Q, Wu B, Jiang C, Wang Y, Xu T. Improving the water dissociation efficiency in a bipolar membrane with amino-functionalized MIL-101. *Journal of Membrane Science* **524**, 370-376 (2017).

19. Shen C, Wycisk R, Pintauro PN. High performance electrospun bipolar membrane with a 3D junction. *Energy & Environmental Science* **10**, 1435-1442 (2017).

20. Manohar M, Shukla G, Pandey RP, Shahi VK. Efficient bipolar membrane with protein interfacial layer for optimal water splitting. *Journal of Industrial and Engineering Chemistry* **47**, 141-149 (2017).

21. Chen G-F, *et al.* Electrochemical reduction of nitrate to ammonia via direct eight-electron transfer using a copper–molecular solid catalyst. *Nature Energy* **5**, 605-613 (2020).

22. Wang Y, Zhou W, Jia R, Yu Y, Zhang B. Unveiling the Activity Origin of a Copper-based Electrocatalyst for Selective Nitrate Reduction to Ammonia. *Angewandte Chemie International Edition* **59**, 5350-5354 (2020).

23. Wu Z-Y, *et al.* Electrochemical ammonia synthesis via nitrate reduction on Fe single atom catalyst. *Nature Communications* **12**, 2870 (2021).

24. Lim J, *et al.* Structure Sensitivity of Pd Facets for Enhanced Electrochemical Nitrate Reduction to Ammonia. *ACS Catalysis* **11**, 7568-7577 (2021).

25. Li P, Jin Z, Fang Z, Yu G. A single-site iron catalyst with preoccupied active centers that achieves selective ammonia electrosynthesis from nitrate. *Energy & Environmental Science* **14**, 3522-3531 (2021).

26. Jia R, Wang Y, Wang C, Ling Y, Yu Y, Zhang B. Boosting Selective Nitrate Electroreduction to Ammonium by Constructing Oxygen Vacancies in TiO2. *ACS Catalysis* **10**, 3533-3540 (2020).

27. McEnaney JM*, et al.* Electrolyte Engineering for Efficient Electrochemical Nitrate Reduction to Ammonia on a Titanium Electrode. *ACS Sustainable Chemistry & Engineering* **8**, 2672-2681 (2020).

28. Li J*, et al.* Efficient Ammonia Electrosynthesis from Nitrate on Strained Ruthenium Nanoclusters. *Journal of the American Chemical Society* **142**, 7036-7046 (2020).

29. Wang Y*, et al.* Enhanced Nitrate-to-Ammonia Activity on Copper–Nickel Alloys via Tuning of Intermediate Adsorption. *Journal of the American Chemical Society* **142**, 5702-5708 (2020).

30. Fan X*, et al.* In situ grown Fe3O4 particle on stainless steel: A highly efficient electrocatalyst for nitrate reduction to ammonia. *Nano Research* **15**, 3050-3055 (2022).

31. Chen Q*, et al.* CoO nanoparticle decorated N-doped carbon nanotubes: a high-efficiency catalyst for nitrate reduction to ammonia. *Chemical Communications* **58**, 5901-5904 (2022).

32. Crawford J, Yin H, Du A, O'Mullane AP. Nitrate‐to‐Ammonia Conversion at an InSn‐Enriched Liquid‐Metal Electrode. *Angewandte Chemie*, e202201604 (2022).

33. Jeon TH, Wu Z-Y, Chen F-Y, Choi W, Alvarez PJJ, Wang H. Cobalt–Copper Nanoparticles on Three-Dimensional Substrate for Efficient Ammonia Synthesis via Electrocatalytic Nitrate Reduction. *The Journal of Physical Chemistry C* **126**, 6982-6989 (2022).

34. Zhao Y, Liu Y, Zhang Z, Mo Z, Wang C, Gao S. Flower-like open-structured polycrystalline copper with synergistic multi-crystal plane for efficient electrocatalytic reduction of nitrate to ammonia. *Nano Energy* **97**, 107124 (2022).

35. Deng X, Yang Y, Wang L, Fu X-Z, Luo J-L. Metallic Co Nanoarray Catalyzes Selective NH3 Production from Electrochemical Nitrate Reduction at Current Densities Exceeding 2 A cm−2. *Advanced Science* **8**, 2004523 (2021).

36. Chen F-Y*, et al.* Efficient conversion of low-concentration nitrate sources into ammonia on a Ru-dispersed Cu nanowire electrocatalyst. *Nature Nanotechnology* **17**, 759-767 (2022).

37. Fang Z, Jin Z, Tang S, Li P, Wu P, Yu G. Porous Two-dimensional Iron-Cyano Nanosheets for High-rate Electrochemical Nitrate Reduction. *ACS Nano* **16**, 1072-1081 (2022).

38. Liu Z, Wang C, Chen C, Li C, Guo C. Selective electroreduction of nitrate to ammonia with high Faradaic efficiency on nanocrystalline silver. *Electrochemistry Communications* **131**, 107121 (2021).

39. Liu C-Y, Sung C-C. A review of the performance and analysis of proton exchange membrane fuel cell membrane electrode assemblies. *Journal of Power Sources* **220**, 348-353 (2012).

40. Ge L, *et al.* Electrochemical CO2 reduction in membrane-electrode assemblies. *Chem* **8**, 663-692 (2022).

41. Sokalski T, Lewenstam A. Application of Nernst–Planck and Poisson equations for interpretation of liquid-junction and membrane potentials in real-time and space domains. *Electrochemistry Communications* **3**, 107-112 (2001).

42. Sokalski T, Lingenfelter P, Lewenstam A. Numerical Solution of the Coupled Nernst−Planck and Poisson Equations for Liquid Junction and Ion Selective Membrane Potentials. *The Journal of Physical Chemistry B* **107**, 2443-2452 (2003).

43. Onsager L. Deviations from Ohm's Law in Weak Electrolytes. *The Journal of Chemical Physics* **2**, 599-615 (1934).

44. Tuckerman ME, Marx D, Parrinello M. The nature and transport mechanism of hydrated hydroxide ions in aqueous solution. *Nature* **417**, 925-929 (2002).

45. Golubenko DV, Yaroslavtsev AB. Effect of current density, concentration of ternary electrolyte and type of cations on the monovalent ion selectivity of surface-sulfonated graft anion-exchange membranes: modelling and experiment. *Journal of Membrane Science* **635**, 119466 (2021).

46. Hohenadel A, Gangrade AS, Holdcroft S. Spectroelectrochemical Detection of Water Dissociation in Bipolar Membranes. *ACS Applied Materials & Interfaces* **13**, 46125-46133 (2021).

47. Blommaert MA, Vermaas DA, Izelaar B, in ’t Veen B, Smith WA. Electrochemical impedance spectroscopy as a performance indicator of water dissociation in bipolar membranes. *Journal of Materials Chemistry A* **7**, 19060-19069 (2019).
