# Supplementary material for: Continuous ammonia electrosynthesis using physically interlocked bipolar membrane at 1000 mA cm−2
Source: Nat Commun. 2023 Mar 23;14:1619. doi: 10.1038/s41467-023-37273-7 (PMC10036611; doi:10.1038/s41467-023-37273-7)
Supplement: Supplementary file 3 — Description to Additional Supplementary Information [file 41467_2023_37273_MOESM3_ESM.pdf]

Description of Additional Supplementary Files

Supplementary Video 1. 3D reconstruction of MBM.

Supplementary Video 2. 3D reconstruction of flat interface BM
